# *In vivo* evidence for an endothelium-dependent mechanism in radiation-induced normal tissue injury

**DOI:** 10.1038/srep15738

**Published:** 2015-10-29

**Authors:** Emilie Rannou, Agnès François, Aurore Toullec, Olivier Guipaud, Valérie Buard, Georges Tarlet, Elodie Mintet, Cyprien Jaillet, Maria Luisa Iruela-Arispe, Marc Benderitter, Jean-Christophe Sabourin, Fabien Milliat

**Affiliations:** 1 Institut de Radioprotection et de Sûreté Nucléaire (IRSN), Department of Radiobiology and Epidemiology (SRBE), Research on Radiobiology and Radiopathology Laboratory (L3R), Fontenay-aux-Roses, 92260, France; 2 Department of Molecular, Cell, and Developmental Biology, University of California, Los Angeles; 3 Institut de Radioprotection et de Sureté Nucléaire (IRSN), Department of Radiobiology and Epidemiology (SRBE), Fontenay-aux-Roses, France; 4 Department of Pathology, Rouen University Hospital, France

## Abstract

The pathophysiological mechanism involved in side effects of radiation therapy, and especially the role of the endothelium remains unclear. Previous results showed that plasminogen activator inhibitor-type 1 (PAI-1) contributes to radiation-induced intestinal injury and suggested that this role could be driven by an endothelium-dependent mechanism. We investigated whether endothelial-specific PAI-1 deletion could affect radiation-induced intestinal injury. We created a mouse model with a specific deletion of PAI-1 in the endothelium (PAI-1KO^endo^) by a Cre-LoxP system. In a model of radiation enteropathy, survival and intestinal radiation injury were followed as well as intestinal gene transcriptional profile and inflammatory cells intestinal infiltration. Irradiated PAI-1KO^endo^ mice exhibited increased survival, reduced acute enteritis severity and attenuated late fibrosis compared with irradiated PAI-1^flx/flx^ mice. Double E-cadherin/TUNEL labeling confirmed a reduced epithelial cell apoptosis in irradiated PAI-1KO^endo^. High-throughput gene expression combined with bioinformatic analyses revealed a putative involvement of macrophages. We observed a decrease in CD68^+^cells in irradiated intestinal tissues from PAI-1KO^endo^ mice as well as modifications associated with M1/M2 polarization. This work shows that PAI-1 plays a role in radiation-induced intestinal injury by an endothelium-dependent mechanism and demonstrates *in vivo* that the endothelium is directly involved in the progression of radiation-induced enteritis.

Used for more than half of patients with tumors, radiotherapy plays a crucial role in cancer cure. The therapeutic index of radiotherapy depends on two parameters, tumor control and normal tissue tolerance. Despite huge advances in the planning of dose distribution to the target volume, toxicity of surrounding healthy tissues remains the most important radiation dose-limiting factor^1^. Tumors in the abdominal cavity and pelvis account for more than half of radiation treatments, and in recent years the notion has emerged of “pelvic radiation disease”, which covers all symptoms associated with healthy tissue toxicity, from acute complications to chronic and fibrotic damage, the latter affecting 10% of patients^2^. Often underestimated, radiation enteropathy is a real clinical problem and long-term prevalence exceeds that of inflammatory bowel disease^3^. If we want to identify relevant therapeutic approaches, the crucial scientific challenge is to improve our knowledge of the pathophysiological mechanisms involved in the progression of radiation enteropathy.

Tissue response to radiation has long been explained by the target cell concept^4^. Concerning radiation enteropathy, the severity of epithelial depletion has long been considered as the sole determinant of acute intestinal injury. The contemporary view involves several cell types and molecular mechanisms, which together form an orchestrated response, and contribute to the initiation, progression and chronicity of radiation-induced injury^3^. The concept that the microvasculature plays a central role in the radiation toxicity of many tissues, including the intestine^5^, is often described, but lacks robust demonstration. Irradiation leads to endothelial cell apoptosis, increased vascular permeability, and acquisition of a pro-inflammatory and pro-coagulant phenotype. These modifications strongly participate in the development of radiation-induced damage, notably in the bowel^6^. We have used tissue-specific knockout models to study the role of the endothelial compartment in the progression of radiation-induced intestinal injury. We hypothesized that the pool of plasminogen activator inhibitor-type 1 (PAI-1) produced by endothelial cells could be involved in the development and progression of radiation-induced intestinal damage. PAI-1 belongs to the family of serine protease inhibitors, and is the main inhibitor of plasminogen to plasmin conversion via inhibition of its targets uPA and tPA^7^. Consequently, PAI-1 limits fibrin degradation and plasmin-dependent matrix metalloproteinase activation. PAI-1 is produced by several cell types in pathological conditions and is involved in many pathophysiological processes, including inflammation^8^, fibrosis^9^,^10^ and macrophage adhesion/migration^11^. So far, it has been shown that PAI-1 is overexpressed in the endothelial cells of different irradiated healthy tissues in patients^12^,^13^. Moreover, PAI-1 genetic deficiency in mice limits the severity of radiation-induced intestinal injury^13^, and improves skin wound healing after irradiation^14^. There is a body of evidence to connect PAI-1 to the endothelial response to radiation and the severity of radiation-induced damage, although this link has not been demonstrated. In the present work, we investigated whether specific PAI-1 deletion in the endothelium affects the intestinal response to radiation exposure, and show that the endothelium is directly involved in the progression of radiation-induced enteritis.

## Results

### Endothelial PAI-1 deletion protects mice against acute radiation-induced intestinal injury

**Figure 1:**
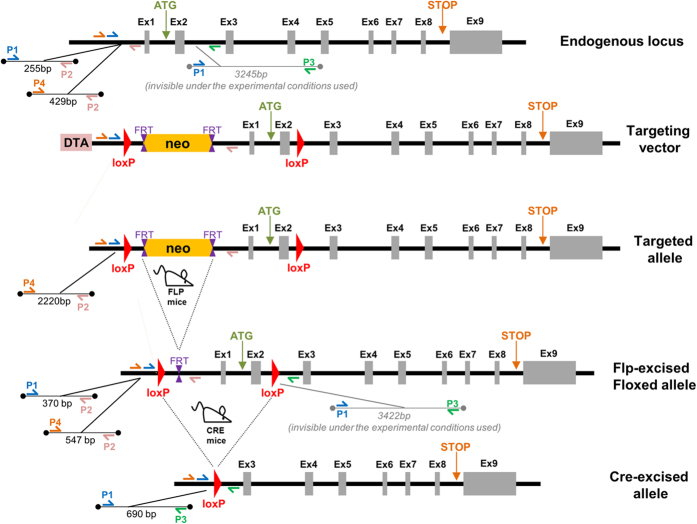
Generation of endothelium-specific PAI-1 knockout mice. Molecular targeting strategy. Primers P1 to P4 used for PCR analysis are indicated on alleles. P1-P2 and P1-P3 products are used for mice genotyping, while P2-P4 products are used for neo-cassette excision checking. ex = exon; DTA = diphtheria toxin A fragment gene; neo = neomycin cassette; FLP = flip-flop recombinase; FRT = Flp recognition target; loxP = locus of X-over P1; Cre = cyclic recombinase; ATG = start codon; STP = stop codon.

**Figure 2:**
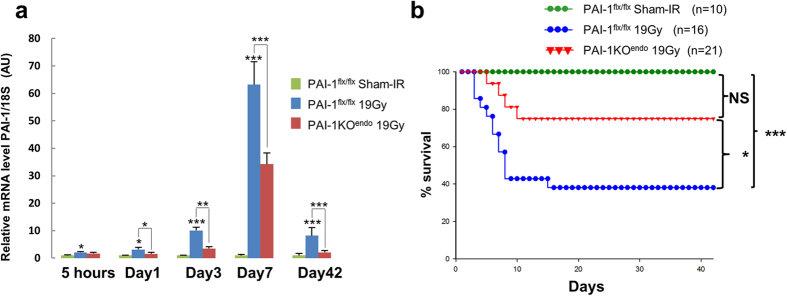
PAI-1 endothelial deletion limits radiation-induced up-regulation of intestinal PAI-1 expression and protects mice from death in a radiation-induced enteritis model. (**a**) Relative PAI-1 mRNA level was measured by RT-qPCR in intestinal tissue in PAI-1^flx/flx^ sham-IR, and in irradiated PAI-1^flx/flx^ and PAI-1KO^endo^ mice. Results are means ± SEM with *P < 0.05,**P < 0.01 and ***P < 0.001 with n = 8 to 12 mice per group. (**b**) Kaplan-Meier analyses representing the percent survival of irradiated PAI-1^(flx/flx)^ mice and PAI-1KO^endo^ mice. The log rank test was used for statistical analyses with NS, non-significant, *P < 0.05 and ***P < 0.001.

**Figure 3:**
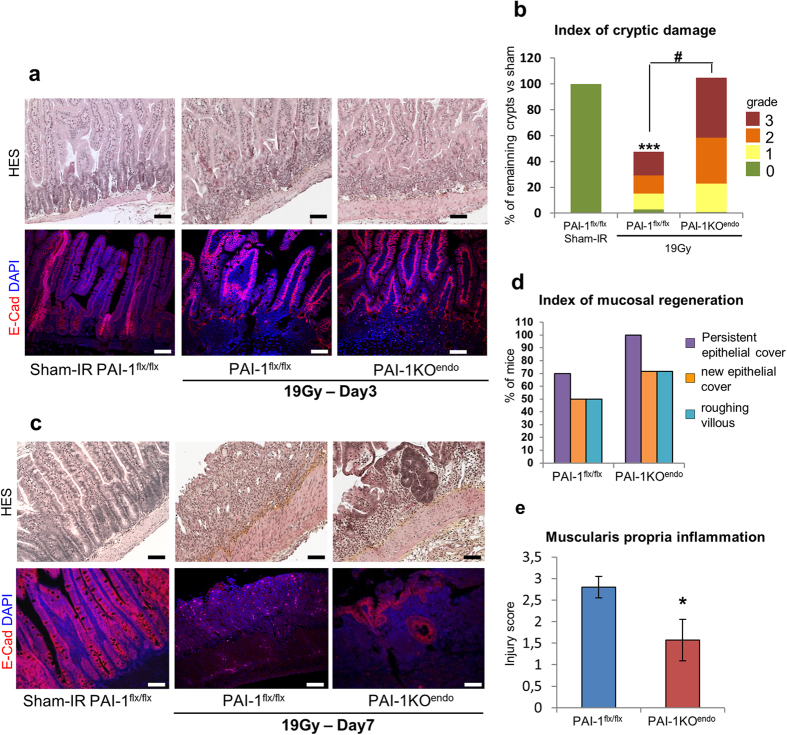
Endothelial-specific PAI-1 deletion limits acute radiation enteritis. (**a**) Representative microscopic alterations obtained in PAI-1^flx/flx^ and PAI-1KO^endo^ 3 days after irradiation. Slides were stained with hematoxylin-eosin-saffron (upper panels) or with antibody against E-cadherin (red) and counterstained with DAPI (blue) (lower panels). Scale bar = 100 μm. (**b**) The number of crypts as well as the severity of cryptic damage were evaluated for each group. The number of crypts is expressed as a percentage of sham-IR mice. ***P < 0.001 versus PAI-1^flx/flx^ sham-IRmice; ^#^P < 0.01 versus PAI-1^flx/flx^/19 Gy mice (8 to 12 mice per group). For each group, crypts are categorized according to severity of their damage. Lesions range from grade 0 (no lesion) to 3 (phantom crypt). Results are expressed as a percentage of total crypts. (**c**) Representative microscopic alterations obtained in PAI-1^flx/flx^ and PAI-1KO^endo^ 7 days after irradiation.(**d**) Parameters of mucosal regeneration were evaluated. Results are expressed as a percentage of mice showing these parameters with 8 to 12 mice per group. (**e**) Evaluation of the severity of muscularis propria inflammation. Scoring ranges from 0 (no lesion) to 4 (loss of muscularis propria). *P < 0.01.

In order to study the consequences of genetic inactivation of PAI-1 in endothelial cells, we generated PAI-1 floxed mice (Fig. 1 and Supplementary Fig. 1a,b) and crossed them with VECad-Cre mice to produce endothelial-specific PAI-1 knockout mice (Supplementary Fig. 2). The specificity of endothelium recombination events in intestinal tissue was checked using ROSA26 reporter mice crossed with VECad-Cre or VECad-Cre^i^ mice (Supplementary Fig. 3 and 4) and a genotyping strategy was used to genotype the mice and to detect the excised allele (Supplementary Fig. 1c). We observed decreased expression of PAI-1 mRNA in lung and gut in PAI-1KO^endo^ mice compared with PAI-1^flx/flx^ mice (Supplementary Fig. 1d). We observed no differences in PAI-1 mRNA level between PAI-1^flx/flx^ mice and C57BL/6J mice (Supplementary Fig. 1e) showing that Loxp sites insertion has no effects on PAI-1 basal level expression. In a model of radiation enteropathy, intestinal PAI-1 expression increased from 5 h to up to 6 weeks post-exposure, while endothelial PAI-1 deletion partially limited this radiation-induced PAI-1 up-regulation (Fig. 2a). We monitored survival and observed that genetic PAI-1 deletion in endothelium protects mice from death after a high dose of ionizing radiation localized to a small part of the gut (Fig. 2b). More than 60% of irradiated PAI-1^flx/flx^ mice died within two weeks (P = 0.0014 versus sham-IR PAI-1^flx/flx^ mice), whereas about 75% of PAI-1KO^endo^ mice survived (P = 0.084 versus sham-IR PAI-1^flx/flx^ mice and p = 0.014 versus irradiated PAI-1^flx/flx^ mice). We examined intestinal tissue injury in depth 3 and 7 days after irradiation (Fig. 3). Mucosal injury was reduced in irradiated PAI-1KO^endo^ mice compared with irradiated PAI-1^flx/flx^ mice (Fig. 3a–c), with a better index of cryptic damage at day 3 (Fig. 3b) and signs of a mucosal regeneration and reduced muscle inflammation at day 7 after irradiation (Fig. 3d,e).

### Radiation-induced epithelial cell death is reduced in PAI-1KO^endo^ mice

**Figure 4:**
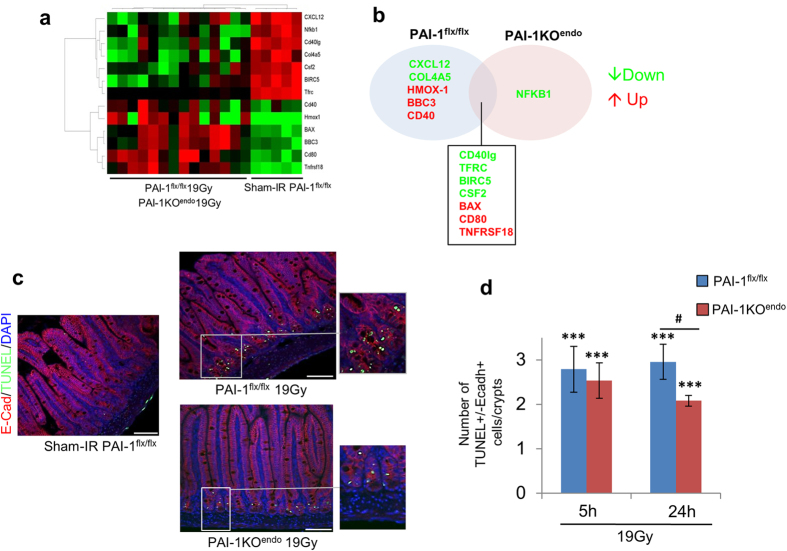
Endothelial-specific PAI-1 deletion reduces acute radiation-induced epithelial cell death. (**a**) Gene expression profiles (5 hours after irradiation) with significant differences between sham-IR and irradiated mice were visualized by a heat map. (**b**) Venn diagram of genes with a significant mRNA level modification in irradiated PAI-1^flx/flx^ and PAI-1KO^endo^ mice compared with the sham-IR group. (**c**) Representative microscopic alterations obtained in PAI-1^flx/flx^ mice and PAI-1KO^endo^ mice, irradiated or not. Slides were double-stained with antibody against E-cadherin (red) and TUNEL labeling (green), then counterstained with DAPI (blue). Scale bar = 100 μm. (**d**) The number of apoptotic cells in crypts was evaluated for each group (n = 6 mice per group). Results are expressed as number of epithelial apoptotic cells per crypt. ***P < 0.001 versus PAI-1^flx/flx^ sham mice; ^#^P < 0.01 versus PAI-1^flx/flx^ 19 Gy.

To explain the differences we observed in the acute phase, we plotted a molecular expression profile 5 h after irradiation. The mRNA levels of 106 genes were measured by real-time PCR using a TaqMan low-density array (TLDA) complemented with a panel of 12 individual genes. Biological information was extracted using both statistical and bioinformatic tools. Hierarchical clustering analyses discriminated sham-IR from irradiated mice (Supplementary Fig. 5a). Statistical analyses revealed a specific molecular signature of radiation exposure according to the expression or not of PAI-1 in the endothelium. This molecular signature is shown in Fig. 4a and Supplementary Fig. 5b–e. We detected similar decreased expression of BIRC5 and increased expression of Bax in both irradiated PAI-1KO^endo^ and PAI-1^flx/flx^ mice (Supplementary Fig. 5f). However, up-regulation of BBC3, a gene that has been implicated in radiation-induced intestinal injury^15^, was only observed in irradiated PAI-1^flx/flx^ mice, suggesting differences in apoptosis-related effects in the 2 mouse lineages. Double labeling of epithelial cells and TUNEL-positive cells revealed that epithelial cell apoptosis in intestinal crypts was significantly increased in irradiated mice 5 h and 24 h after irradiation, whatever the status of PAI-1. However, the level of apoptotic cells was reduced in irradiated PAI-1KO^endo^ mice compared with irradiated PAI-1^flx/flx^ mice (Fig. 4c,d).

### Constitutive and inducible endothelial PAI-1 deletions protect mice from late radiation-induced intestinal injury

**Figure 5:**
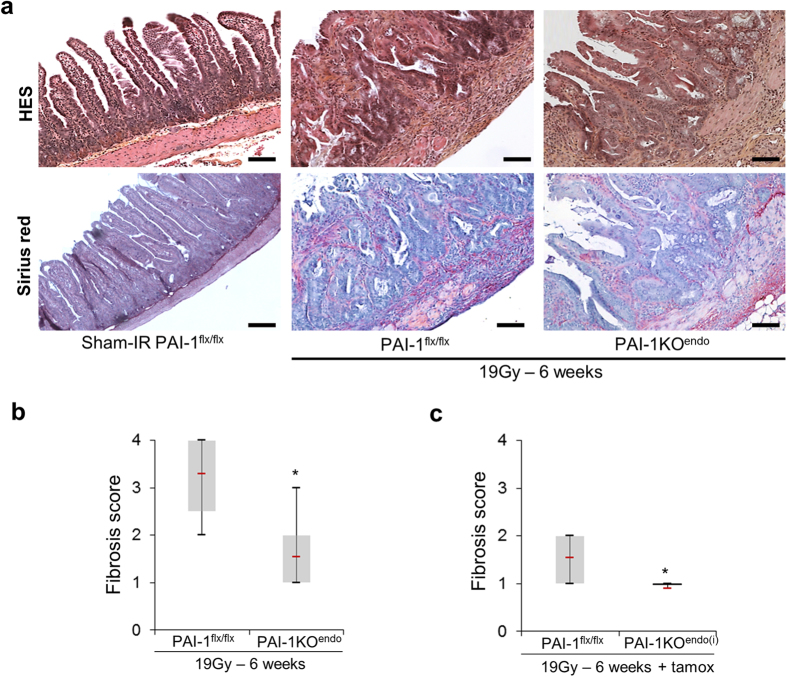
Constitutive and inducible endothelial-specific PAI-1 deletion limits fibrosis following a single high-dose radiation exposure. (**a**) Representative microscopic alterations obtained in PAI-1^flx/flx^ mice and PAI-1KO^endo^ mice 6 weeks after irradiation. Slides were stained with hematoxylin-eosin-saffron (upper panels) or Sirius red (lower panels). Scale bar = 100 μm n = 5 for PAI-1^flx/flx^sham-IR mice; n = 8 for other groups. Fibrosis score in constitutive (**b**) or inducible (**c**) PAI-1KO^endo^ mice (named PAI-1KO^endo(i)^). Scores ranged from 0 (no damage) to 4 (severe fibrosis). All sham-IR mice displayed a score of 0 (not shown). For experiments with inducible mice, the 3 groups were treated in the same conditions with tamoxifen. n = 5 for PAI-1^flx/flx^ sham-IR mice (scores of 0 are not shown) and n = 8 to 11 for the other groups. *P < 0.05.

As described by Zheng *et al.*^16^, the model of localized intestinal radiation injury offers the opportunity to study the progression of damage over several weeks. Six weeks after irradiation, we observed patches of intestinal fibrosis, as shown in a previous study by our team^13^. Sirius red staining revealed that collagen deposition is reduced in irradiated PAI-1KO^endo^ mice compared with irradiated PAI-1^flx/flx^ mice, as reflected by the fibrosis score (Fig. 5a,b). To confirm these results we also used an inducible knockout model using VECad-creER^T2^ mice. We observed a reduced radiation-induced fibrosis score in PAI-1KO^endo(i)^ mice compared with PAI-1^flx/flx^ mice treated with tamoxifen (Fig. 5c).

### Endothelial PAI-1 deletion impacts the intestinal gene expression profile following radiation exposure

**Figure 6:**
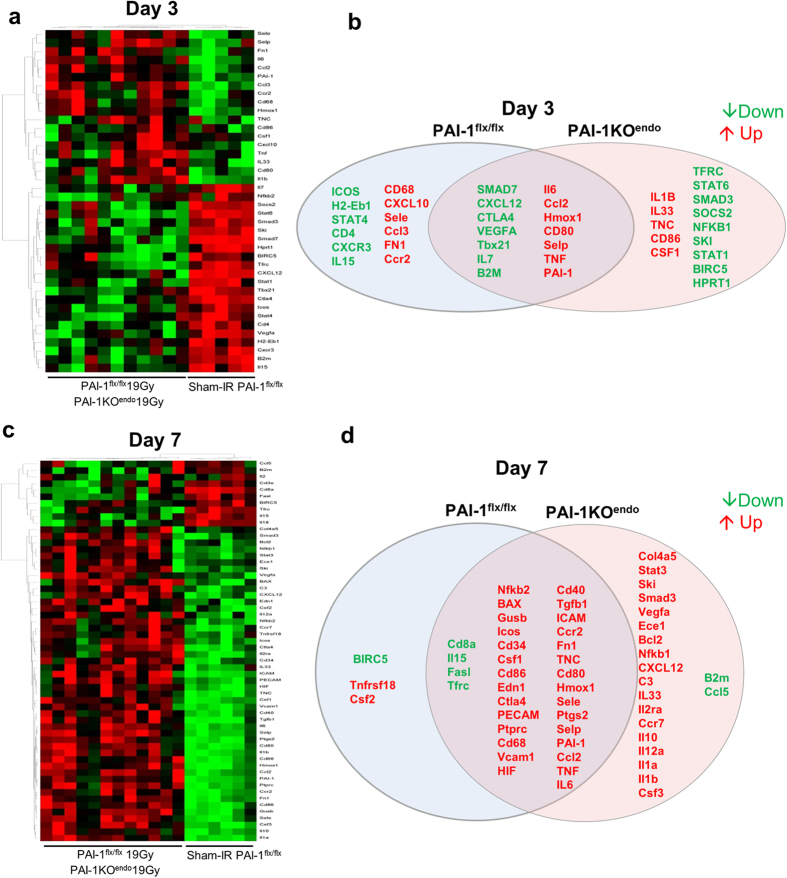
Endothelial-specific PAI-1 deletion impacts the molecular profile associated with immune-related genes in irradiated intestinal tissue. Gene expression profiles 3 days (**a**) and 7 days (**b**) after irradiation showing significant differences between sham-IR and irradiated mice are visualized in the heat map. (**c**,**d**) Corresponding Venn diagrams of genes with a significant change in mRNA level in irradiated PAI-1^flx/flx^ mice and PAI-1KO^endo^ mice compared with the sham-IR group.

Because PAI-1 has anti-fibrinolytic properties, we hypothesized that reduced acute and late intestinal injury in PAI-1KO^endo^ mice could be due to differences concerning fibrinolysis. We observed acute and chronic fibrin deposition in irradiated animals, but no differences between the 2 genotypes were noted (Supplementary Fig. 6), suggesting that the differences between the mouse lineages did not depend on fibrinolysis. Since the difference between the 2 lineages could be explained by a difference in the immune response, we next investigated the immune gene expression profile at day 3 and day 7 after irradiation by RT-qPCR using a TLDA methodology complemented with individual qPCR (Supplementary Figs 7 to 10). As for the 5-h time-point, hierarchical clustering analyses put sham-IR and irradiated mice into 2 different clusters, while the 2 mouse lineages PAI-1^flx/flx^ and PAI-1KO^endo^ could not be differentiated by this unsupervised statistical analysis tool (Fig. 6a–c and Supplementary Figs 7a and 9a). In-depth analyses of these results using supervised statistics and bioinformatic tools revealed differences between irradiated PAI-1^flx/flx^ and PAI-1KO^endo^ mice at both 3 and 7 days after irradiation (Fig. 6b–d). Volcano plots identified a specific radiation signature according to PAI-1 status in the endothelium (Supplementary Fig. 7b,c and Supplementary Fig. 9b,c). Bioinformatic tools were then used to establish whether a particular biological function could explain the protection of PAI-1KO^endo^ mice from radiation-induced damage (Supplementary Fig. 8 and 10, and Supplementary Table 1). Interestingly, gene ontology enrichment analyses revealed clear differences between PAI-1^flx/flx^ and PAI-1KO^endo^ mice following radiation exposure (Supplementary Table 2). According to the total number of entities in each enrichment result, the “response to hypoxia” Gene Ontology (GO) term was ranked first in the irradiated PAI-1 KO^endo^ mouse group, but was not ranked in the irradiated PAI-1^flx/flx^ mouse group. These results led us to examine whether PAI-1 up-regulation could be driven by a hypoxia-dependent molecular mechanism. We therefore generated mice with a specific genetic hypoxia inducible factor-1α (HIF-1α) deletion in endothelial cells (VECad-Cre^+/−^/HIF-1α^flx/flx^), allowing us to show that intestinal PAI-1 overexpression after local intestine irradiation is at least partly dependent on HIF-1α expression in the endothelium (Supplementary Fig. 11). We detected overexpression at 3 and 7 days of several molecules involved in the positive chemotaxis of both neutrophils and monocytes (CCL2, TNF, VEGFA, IL6, CCL3) (Fig. 6 and Supplementary Table 1), indicating that this process could be important in the observed phenomenon. Myeloperoxidase (MPO) labeling showed no differences between irradiated mice, whatever the status of PAI-1 in the endothelium (Supplementary Fig. 12). Moreover, we observed that TNFα overexpression was higher in irradiated PAI-1^flx/flx^ mice (fold change of 17.4) than in irradiated PAI-1KO^endo^ mice (fold change of 6.9) at day 7 post-exposure, compared with sham-IR mice (Supplementary Table 1). On the other hand, 3 days after irradiation, the macrophage marker CD68 gene was only overexpressed in PAI-1^flx/flx^ mice. Seven days after irradiation, CD68 overexpression was almost two times higher in irradiated PAI-1^flx/flx^ mice than in irradiated PAI-1KO^endo^ mice (fold changes of respectively 8.7 and 4.9) (Supplementary Table 1). Altogether, these results suggest that PAI-1 deletion in endothelium affected radiation-induced macrophage infiltration.

### Conditional endothelium-specific PAI-1 deletion limits macrophage infiltration and influences macrophage M1/M2 polarization

**Figure 7:**
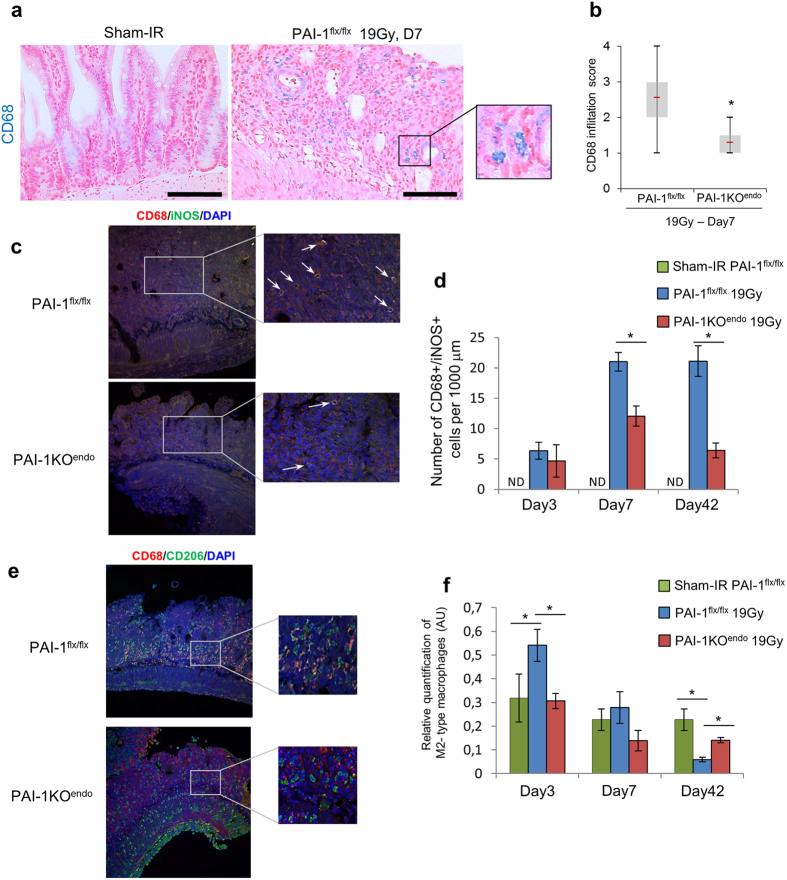
Conditional endothelium-specific PAI-1 deletion limits macrophage infiltration and influences macrophage M1/M2 polarization. (**a**) Representative labeling of macrophages in intestinal tissue 7 days after irradiation. Slides were stained with antibodies against CD68 (blue) and counterstained with nuclear fast red (pink). Scale bar = 100 μm. (**b**) Macrophage scoring. Scores ranged from 0 (sham-IR) to 4 (maximum macrophage count). n = 6 for sham-IRPAI-1^flx/flx^mice, n = 8 for PAI-1^flx/flx^ 19 Gy mice, and n = 6 for PAI-1KO^endo^19 Gy mice. *P < 0.01; (**c**) Representative double labeling of M1 macrophages in intestinal tissue 1 week after irradiation. Slides were stained with antibodies against CD68 (red) and iNOS (green) and counterstained with DAPI. (**d**) Quantification of M1 macrophages (yellow merging signal) in sham-IR PAI-1^flx/flx^mice, PAI-1^flx/flx^ 19 Gy mice and PAI-1KO^endo^19 Gy mice at 3, 7 and 42 days after irradiation. *P < 0.05 ND: not detected in sham-IR mice. (**e**) Representative double labeling of M2 macrophages in intestinal tissue 7 days after irradiation. Slides were stained with antibodies against CD68 (red) and CD206 (green) and counterstained with DAPI. (**f**) Quantification of M2 macrophages (yellow merging signal) in sham-IR PAI-1^flx/flx^ mice, PAI-1^flx/flx^19 Gy mice and PAI-1KO^endo^19 Gy mice at 3, 7 and 42 days after irradiation. For all experiments, n = 6 for sham-IRPAI-1^flx/flx^mice, n = 8 for PAI-1^flx/flx^19 Gy mice, and n = 6 for PAI-1KO^endo^19 Gy mice. *P < 0.05.

We monitored macrophage infiltration and polarization during the progression of radiation enteropathy. Seven days after irradiation, immunolabeling experiments showed a decrease of CD68+ cells in irradiated PAI-1KO^endo^ mice compared with irradiated PAI-1^flx/flx^ mice (Fig. 7a,b). A slight decrease of CD68+ cells was also observed at 3 days, but there were no differences after 6 weeks (Supplementary Fig. 13). Because macrophage polarization is a crucial process involved in wound healing, we next monitored macrophage polarization using CD68/iNOS and CD68/CD206 double immunolabeling to quantify the levels of M1 and M2 macrophage polarization (Fig. 7c,e). The number of M1 macrophages increased following radiation exposure, at 3, 7 and up to 42 days after irradiation (Fig. 7d). However, the increased level of M1 cells was reduced in irradiated mice with endothelial PAI-1 genetic deletion. For M2 polarization, we observed at 3 days an increased level of CD68/CD206+ cells in irradiated PAI-1^flx/flx^ mice, but not in PAI-1KO^endo^ mice (Fig. 7f). While no statistical difference was noted at day 7 between the 2 mouse strains, we observed that the level of M2 macrophages was higher in PAI-1KO^endo^ mice than in PAI-1^flx/flx^ mice 6 weeks after irradiation (Fig. 7f).

## Discussion

This work strengthens the concept that endothelium strongly contributes to the progression of radiation-induced intestinal injury. Using a new model of transgenic mice specifically knocked-out for PAI-1 in endothelial cells, we demonstrate that this protein orchestrates the progression of enteritis by an endothelium-dependent mechanism.

Endothelium has already been described as a crucial compartment involved in gastrointestinal syndrome (GIS)^17^,^18^,^19^ in studies that used total body or abdominal irradiation. However, conflicting results obtained with intravascular boronated liposome have challenged this concept^20^,^21^ and the role of endothelium in normal tissue radiation injury need to be cleared. Unlike GIS models, the model of radiation enteropathy that we used allows exploration of the progression of enteritis and radiation-induced late effects. Aware that a single dose of 19 Gy is not representative of or comparable to the conventional fractionation scheme used in clinical practice, this preclinical model is nonetheless useful in providing proof of principle that a specific molecular target in a specific compartment may be associated with radiation injury. Moreover, the tendency of radiation therapy practice to move toward high doses per fraction, such as in stereotactic body radiation therapy for prostate cancer, raises the question of potentially enhanced injury to organs at risk^22^. Clearly, this preclinical model could help to address some scientific issues in this context.

Advances in genetic engineering provide a powerful model system to study the mechanisms of normal tissue injury after irradiation^23^. In this way, using the Cre-loxP system to delete p53, it was shown that p53 functioned in endothelial cells to protect mice from myocardial injury after whole-heart irradiation^24^. Moreover, using Villin–Cre mice, one study demonstrated that p53 is required in epithelial cells to prevent GIS^25^. We previously showed that PAI-1 total knockout mice are protected against radiation enteritis, but there was no evidence that this was dependent on the PAI-1 endothelial pool^13^. We therefore created PAI-1 floxed mice to answer this question and we present here the first report using this transgenic model. To our knowledge, ours is the first report to demonstrate that conditional specific inactivation of one gene in the endothelium impacts global intestinal response following radiation injury.

PAI-1 is an anti-fibrinolytic and pro-fibrotic protein^7^. Here, we show that irradiation very rapidly induces fibrin deposition. Surprisingly, PAI-1 deletion in endothelium does not affect fibrin deposition, suggesting that PAI-1 contributes to intestinal injury independently of its anti-fibrinolytic action, or that another cellular pool of PAI-1 is involved.

Crosstalk between thrombosis and inflammation is an emerging concept explaining tissue homeostasis following stress or a wound healing process^26^. Relationships between PAI-1 and the inflammatory process have already been described. PAI-1 knockout mice have a lower influx of neutrophils in a model of lung^27^ or renal^28^ injury. Moreover, PAI-1 inhibits neutrophil efferocytosis^29^ and limits spontaneous or TNF-related apoptosis-inducing ligand (TRAIL)-dependent neutrophil apoptosis^30^. In our present work, neutrophil influx was measured using MPO labeling. The results show that endothelial inactivation of PAI-1 does not affect the severity of intestinal neutrophil influx in the acute and late phases after irradiation. This result suggests that the endothelial PAI-1 radiation-induced overexpressed pool is not directly involved in neutrophil influx after irradiation. Fibrinolysis regulators are involved in adhesion of monocytes to endothelial cells *in vitro*. PAI-1 and uPA are required for GlyLDL-induced monocyte adhesion to endothelial cells^31^. Macrophages are highly heterogeneous cells that can adapt their functions in response to local microenvironmental signals.

In our work, gene expression profiles revealed that radiation exposure induces molecular alterations compatible with modification of macrophage polarization. TNFα but also IL6, IL10, Ccr7, Hmox1 and IL1-β have been described to be involved in M1 or M2 polarization and to be expressed by different macrophages subsets^32^. The protective and pathogenic functions of macrophage subsets in the fibrosis and wound healing processes after irradiation are unclear. Macrophage polarization is a mechanism that is dependent on context, such as both the tissue microenvironment and progression of the wound healing process^33^. M1 macrophages are considered as pro-inflammatory immune cells which can exacerbate the inflammatory response by recruiting T helper type 17 lymphocytes and neutrophils, leading to persistent pro-inflammatory signals and substantial tissue damage. In contrast to the pro-inflammatory response triggered by M1 macrophages, M2 macrophages exhibit mainly anti-inflammatory actions. M2 macrophages antagonize the response of M1 macrophages, which may be crucial for the activation of the wound healing process and for restoration of tissue homeostasis. We showed that conditional endothelium-specific PAI-1 deletion limits radiation-induced macrophage infiltration (CD68+ cells) in the radiation acute phase. Levels of M1-type cell influx were also reduced in PAI-1KO^endo^ in the acute and late phases. Although a reduced number of M2-type cells was observed at day 3 after irradiation, a higher number was observed in the late phase in PAI-1KO^endo^ mice compared with floxed irradiated mice. Interestingly, this increase is associated with reduced tissue injury. Further experiments are needed to explore the putative causal links between these two observations and to indicate if macrophages polarization impacts the progression of radiation-induced intestinal injury.

In this work we used VE-cadherin Cre-recombinase mice and showed that PAI-1 deletion in PAI-1KO^endo^ mice impacts immune cell influx. The origin of these immune cells is unknown but a myeloid contribution is probable. Using VEcad-Cre-ROSA26R mice, Alva *et al.* showed that about 50% of all hematopoietic lineages were positive for LacZ in the adult bone marrow^34^. Therefore, we cannot exclude that protection from radiation injury associated with PAI-1 deletion using VEcad-Cre could be due, at least in part, to recombination events in the bone marrow, leading to PAI-1 genetic inactivation in some myeloid progenitors. Interestingly, using VECad-CreER^T2−^ROSA26R mice, Monvoisin *et al.* reported only 0.3% of LacZ+ cells in the bone marrow of adult mice, showing that recombination events in myeloid progenitors are minor events in this model^35^. We confirmed protection from radiation-induced intestinal injury using PAI-1KO^endo^ inducible mice created by crossing VECad-Cre-ER^T2^ tamoxifen inducible Cre mice with PAI-1^fl/fl^ mice. These results confirm that specific PAI-1 endothelial deletion conferred protection against radiation enteritis.

Bioinformatic tools are useful in exploring and analyzing large amounts of data. Here, we measured about one hundred genes in 3 groups of mice at several time points after irradiation, representing thousands of real-time PCR data. We used a pathway analysis tool to explain in detail the differences between mice according their PAI-1 expression in the endothelium. GO enrichment analyses revealed possible differences between mice linked to the response to the hypoxia pathway. Hypoxia response elements are present in the PAI-1 gene promoter and the transcription factor HIF-1α has been shown to be involved in PAI-1-dependent transcription *in vitro*^36^. We therefore hypothesized that HIF-1α could be involved in the radiation-induced PAI-1 up-regulation. Using VECad-Cre^+/−^/HIF-1α^flx/flx^, we have shown here that PAI-1 overexpression is at least in part dependent on HIF-1α expression in endothelium. These results suggest that a hypoxia-PAI-1 axis could be crucial in the progression of radiation-induced enteritis through the endothelium compartment. The detailed mechanisms are not yet fully understood and further experiments are needed to explore them.

In conclusion, we demonstrate in this work that PAI-1 plays a role in the initiation of radiation-induced intestinal injury by an endothelium-dependent mechanism. The endothelial pool of PAI-1 directly or indirectly influences the *in vivo* inflammatory process by affecting recruitment and polarization of macrophages. Our study confirms that PAI-1 is an attractive therapeutic target in attempts to reduce radiation-induced normal tissue injury. We previously tested the PAI-1 inhibitor tiplaxtinin, which had a small beneficial effect by conferring temporary protection against early lethality^37^. Tiplaxtinin inhibits free PAI-1, but not the vitronectin-bound pool of PAI-1^38^, thus limiting *de facto* the efficacy of this PAI-1 inhibitor. New PAI-1 inhibitors have been described^39^,^40^,^41^ recently and should be tested in the light of our results. More conceptually, this work supports the concept that a modification of endothelium phenotype affects the progression of radiation-induced radiation enteritis.

## Materials and Methods

### Generation of PAI-1 floxed mice and animals

The global molecular strategy for creating PAI-1 floxed mice is summarized in Fig. 1. The targeting vector was created from SERPINE1/PAI-1 genomic sequences, which were isolated by PCR amplification of genomic DNA. This vector was linearized by restriction digestion with Fse I, electroporated into 129/Sv ES cells and the transformed cells were subjected to G418 selection. Of 322 G418-resistant ES cell clones, homologous recombination was confirmed in 6 by both Southern blot analysis using 2 different probes outside the region of homology, and PCR analysis with N1 and N2 primers (Supplementary Fig. 1). Three of these clones were used to generate chimeras by standard procedures. Germline transmission was obtained by crossing the chimeras with C57BL/6J females. Heterozygous females were crossed with CMV-Flp males to excise the neomycin selection cassette. When excision of the neomycin selection cassette was successful, a 547-bp PCR product was amplified, using primers P2 and P4 from the genomic tail DNA of the offspring (Fig. 1). Homozygous floxed mice were finally obtained by interbreeding F2 heterozygous floxed mice. The following mice were used for this study: VE-cadherin-Cre (VECad-Cre) mice^34^, VE-cadherin-Cre-ER^T2^ (VECad-Cre^**i**^) mice^35^, ROSA26R LacZ reporter (ROSA) mice (Jackson Laboratory), HIF-1α floxed mice (HIF-1α^flx/flx^) (Jackson Laboratory), and PAI-1 floxed (PAI-1^flx/flx^) mice. Crossing of these lines was used to obtain the following mice: VECad-Cre^+/−^/ROSA^+/+^, VECad-Cre^i +/−^/ROSA^+/+^, VECad-Cre^+/−^/HIF-1α^flx/flx^, VECad-Cre^+/−^/PAI-1^flx/flx^ (PAI-1KO^endo^) and VECad-Cre^i+/−^/PAI-1^flx/flx^ (PAI-1KO^endo(i)^) mice.

### Genotyping of mice

Genomic tail DNA was analyzed by PCR. For genotyping wild-type, targeted, and recombined PAI-1 alleles, 3 primers were used: P1: 5′-CCATGTGGGGAGTCAGACATGCTTC-3′ forward; P2: 5′-CAGCCATCACAGAGAAGCTATGGACC-3′ reverse; P3: 5′-CCAGGCAGATGAGGCTCTTCCAATC-3′ reverse. P1 and P2 detect the wild-type endogenous allele (255 bp) and the floxed allele (370 bp), whereas P1 and P3 detect Cre-excised allele (690 bp) (Fig. 1A). For detection of full excision neomycin cassette alleles, two primers were used: P2 and P4: 5′-GCTGTACTGGTTCTTGCTCCTTGACAGA-3′ forward. A 547-bp PCR product was detected with P1 and P4 when the Flp-mediated excised allele occurred (Fig. 1A). Presence or absence of Cre recombinase was assayed with 3 primers: C1: 5′- GCAGGCAGCTCACAAAGGAACAAT-3′ forward, C2: 5′-TGTCCTTGCTGAGTGACAGTGGAA-3′ reverse, C3: 5′-ATCACTCGTTGCATCGACCGGTAA-3′ reverse. C1 and C2 detect the endogenous VE-cadherin locus (therefore absence of Cre) (550 bp), whereas C1 and C3 detect VE-cadherin-Cre recombinase (310 bp). Presence or absence of the ROSA26 fragment was assayed with three primers: R1: 5′-AAAGTCGCTCTGAGTTGTTAT -3′ forward, R2: 5′-GCGAAGAGTTTGTCCTCAACC-3′ reverse, R3: 5′-GGAGCGGGAGAAATGGATATG -3′ reverse. R1 and R2 detected the ROSA26 fragment (603 bp), whereas R1 and R3 detected the endogenous locus (therefore absence of ROSA26 fragment) (340 bp).

### Experimental procedures

Experiments were conducted in compliance with legal regulations in France for animal experimentation, and protocols were approved by the national ethics committee for animal experimentation of the Institute for Radiological Protection and Nuclear Safety no. 81 (Protocol *13–18*). Radiation enteropathy was induced by exposure of an intestinal segment to 19 Gy of radiation as previously described^13^. Briefly, control PAI-1^flx/flx^ mice and PAI-1KO^endo^ mice were anesthetized with isoflurane and, after laparotomy, a 3 cm-long intestinal segment (10 cm from the ileocecal valve) was exteriorized and exposed to a single dose of 19 Gy of gamma irradiation (^60^Co source, dose rate 0.8 Gy/minute). Sham-irradiation (Sham-IR) was performed by maintaining the intestinal segment exteriorized without radiation exposure. After radiation exposure or sham-irradiation, the exposed segment was returned to the abdominal cavity and peritoneum/abdominal muscles and skin were separately closed with interrupted sutures. Each animal was used for all experiments described below. Activation of CreER^T2^ recombinase was induced by daily intraperitoneal injections of 2 mg tamoxifen (diluted in 10% EtOH in sunflower oil) for 5 days^35^. Irradiations occurred one week after the first injection, a time point at which we checked that CreER^T2^ recombinase was functional.

### Histology and immunohistochemistry

A part of the intestinal segment was assessed by histological examination and immunohistochemistry. Longitudinal pieces were fixed in 4% formaldehyde solution and embedded in paraffin. 5 μm sections were stained with hematoxylin-eosin-saffron and Sirius red. For β-Gal staining, a part of the intestinal tissues was embedded with Tissue-Tek OCT mounting media and frozen in isopentane cooled by liquid nitrogen. Assays were performed on 16 μm frozen sections, using the β-Gal staining kit (Invitrogen) according to the manufacturer’s instructions. Slides were then counterstained with nuclear fast red (Sigma) according to the manufacturer’s instructions. We used the following primary antibodies for immunohistochemistry: rabbit anti-human von Willebrand factor from DAKO, rabbit anti-human fibrinogen from DAKO, rabbit anti-mouse MPO from Abcam, rabbit anti-mouse CD68 from Abcam, rabbit anti-mouse CD206 from Abcam, rabbit anti-mouse iNOS from Abcam and anti-rat E-cadherin (Clone ECDD2) from Life Technology. Goat anti-rabbit Alexa fluor^568^, goat anti-rat Alexa fluor^568^ and goat anti-rabbit Alexa fluor^488^(Molecular Probes) were used as secondary antibodies for immunofluorescent labeling. ImmPress Reagent anti-rabbit Ig (Vector Labs) and Histogreen (Abcys) were used for visible IHC labels.

For fluorescent labeling, all images were recorded using a Zeiss LSM 780 confocal microscope. For E-cadherin/TUNEL double staining, TUNEL staining was performed using the *In Situ* Cell Death Detection Kit (Roche Applied Science) according to the manufacturer’s instructions. Epithelial cells and apoptotic epithelial cells were counted in about 60 crypt sections per sample from the same animals.

Semi-quantitative fibrin deposition score was determined by two authors in a blinded manner and ranged from 0 (no deposition) to 4 (strong deposition). Discrepancies were resolved by discussion.

For immune cell staining (CD68 and MPO), scoring was determined according to the number of cells present in the tissue. Following a first reading, a score was attributed to each animal, ranging from 0 (sham-IR mice) to 4 (maximum number of observed cells) or 2 (minimum number of observed cells). Score was determined in a blinded manner.

M1 type macrophages (CD68+/iNOS+) were quantified as follows. For each mouse, three images were recorded using a Zeiss LSM 780 confocal microscope and double-labeled cells were counted. For each image, the length of intestine was determined and the results were presented as the number of (CD68+/iNOS+)/length unit (here 1000 μm).

M2 type macrophages (CD68+/CD206+) were quantified as follows. For each mouse, three images were recorded using a Zeiss LSM 780 confocal microscope and for each color (red for CD68 or green for CD206) a threshold was determined and fixed. Images were processed using Zen software for automatic quantification of the pixel number of each color or both of them. Results were presented as relative quantification of M2 type macrophages, which represent the ratio between the number of pixels with the two colors and the number of pixels corresponding only to the red (CD68).

**RNA isolation, reverse transcription, real-time qPCR, TLDA and data analysis.**  Total RNA was prepared with the total RNA isolation kit (Rneasy Mini Kit; Qiagen). After quantification on a NanoDrop ND-1000 apparatus (NanoDrop Technologies), 1 μg of RNA was used for reverse transcription with the High Capacity Reverse Transcription Kit (Applied Biosystems) according to the manufacturer’s instructions. Pre-developed TaqMan Gene Expression Assays and TaqMan Mouse Immune Array (Applied Biosystems) were used according to the manufacturer’s instructions. PCR was performed with the ABI PRISM 7900 Sequence detection system (Applied Biosystems). PCR fluorescent signals were normalized to a PCR fluorescent signal obtained from the housekeeping gene 18S. Relative mRNA quantification was performed by using the comparative ΔΔCT method. For each time point, analyses were conducted according to the following procedure. Ct values were extracted from a global analysis using RQ Manager software (Applied Biosytems) in order to apply and normalize optimal baselines and threshold parameters for each target. A text file was extracted and Ct values were imported in Data Assist software (v3.01) in which each mouse was annotated according to its group (Sham-IR, Irradiated Floxed or irradiated PAI-1 KOendo mice). For determining expression fold changes, a maximum allowable Ct value at 37 was fixed and maximum Ct values were not included in calculations. For all analyses, the reference sample group was the Sham-IR group, automatically leading the mean of the reference group to the value 1. The Ct values were normalized using a global normalization method: the software first finds the common assays among all samples and then used the median CT of those assays as the normalizer, on a per sample basis^42^. p-values were adjusted using the Benjamini-Hochberg false discovery rate (FDR) method. Volcano plots were created and used to select the differentially expressed genes using a fold change cut-off of 1.5 and adjusted p-values < 0.05. For unsupervised hierarchical clustering analyses and heat map creation, distances between samples were calculated for hierarchical clustering based on the ΔCT values using Pearson’s correlation, assay centric as map type, and average linkage as clustering method. For each assay, the middle expression level is set as the median of all of the ΔCT values from all samples for that assay. Data points for a given assay can only be compared relative to other data points for that assay. For each map type, the ΔCT value of the neutral/middle expression level (median) is set such that red indicates an increase with a ΔCT value below the middle level, and green indicates a decrease, with a ΔCT value above the middle level. Pathway Studio 10.0 along with ResNet 11.0 from Elsevier, the database of functional relationships and pathways of mammalian proteins (www.elsevier.com/pathway-studio), was used for pathway analysis and gene ontology enrichments.

### Statistical analysis

Data are given as means +/− SEM. Statistical analyses were performed by analysis of variance with a level of significance of p < 0.05. Mouse survival curves were calculated by the Kaplan Meier method and compared using the log rank test.

## Additional Information

**How to cite this article**: Rannou, E. *et al.* *In vivo* evidence for an endothelium-dependent mechanism in radiation-induced normal tissue injury. *Sci. Rep.* **5**, 15738; doi: 10.1038/srep15738 (2015).

## Supplementary Material

Supplementary Information
