# Supplementary material for: In vivo evidence for an endothelium-dependent mechanism in radiation-induced normal tissue injury
Source: Sci Rep. 2015 Oct 29;5:15738. doi: 10.1038/srep15738 (PMC4625166; doi:10.1038/srep15738)
Supplement: Supplementary Information [file srep15738-s1.pdf]

Supplementary information

In vivo evidence for an endothelium-dependent mechanism in radiation-induced normal tissue injury.

Emilie Rannou, Agnès François, Aurore Toullec, Olivier Guipaud, Valérie Buard, Georges Tarlet, Elodie Mintet, Cyprien Jaillet, Maria Luisa Iruela-Arispe, Marc Benderitter, Jean-Christophe Sabourin and Fabien Milliat

Supplementary Figures S1 to S13

Supplementary Table 1 and 2

Supplementary Figure 1

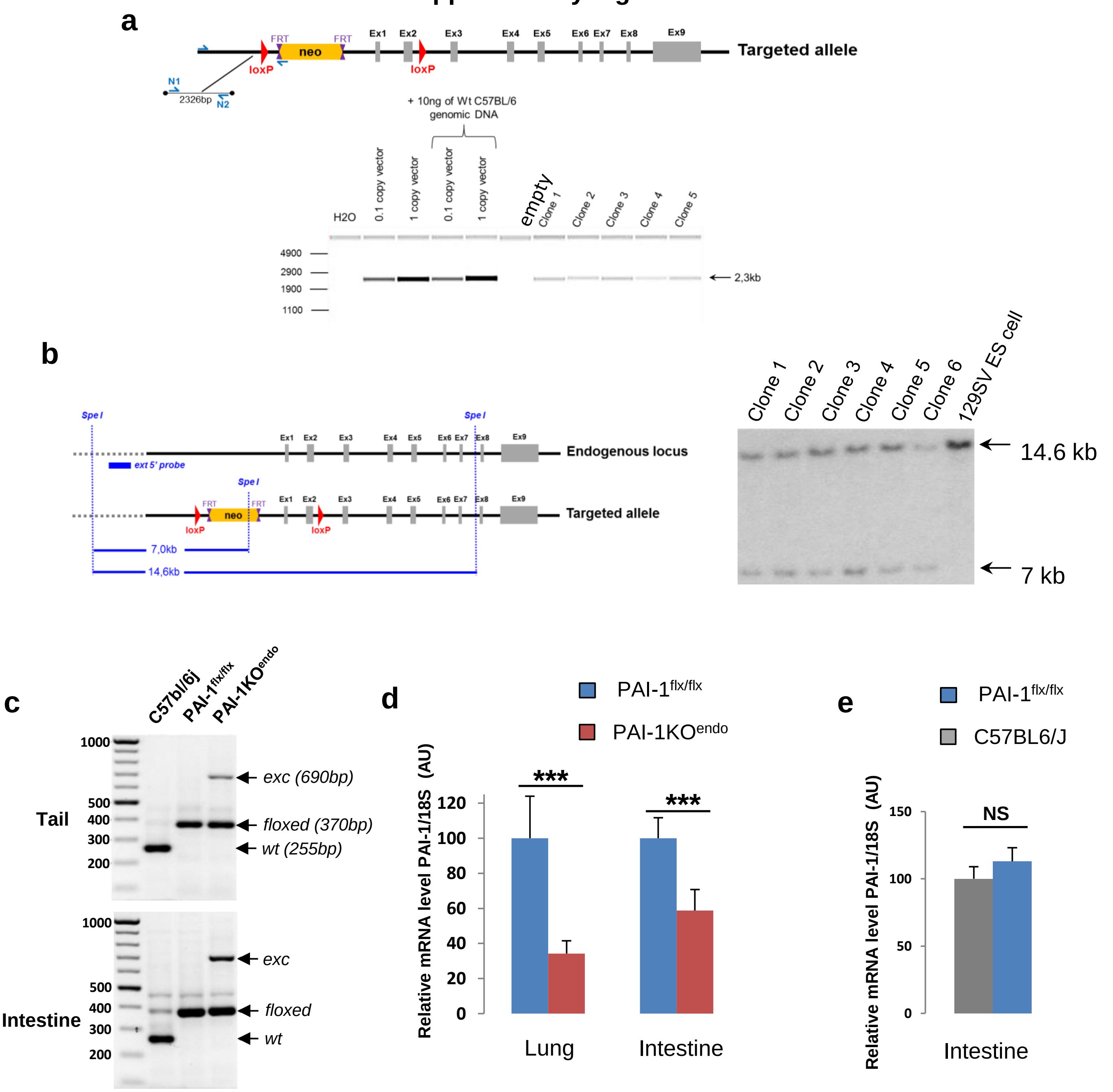

Supplementary Figure 1: Generation of PAI-1 conditional Knockout mice. (**a**) Representative screening of G418-resistant ES cell clones. The 2.3 kb band, using primers N1 and N2, represents the targeted allele. Targeted vectors were used as positive control with two quantities loaded 0.1 or 1 copy alone or in the presence of 10 ng of C57BL/6 genomic DNA, which serves as a negative control. (**b**) A representative southern blot of six G418-resistant ES clones digested with Spe I is shown. The 14.6 kb band for the endogenous allele and the 7 kb band for the targeted allele are indicated. 129SV ES cell serves as a negative control for the targeted allele. (**c**) Genotype identification from DNA tail by PCR to discriminate wild-type, floxed (floxed) and excised (exc) PAI-1 alleles. Genotype was also checked in gut. (**d**) Relative PAI-1 mRNA level was measured by RT-qPCR in lung and intestinal tissue in PAI-1(flx/flx) and PAI-1KOendo mice (n=10 mice per group). Results are means ± SEM; ***, t-test P<0.01. (**e**) Relative PAI-1 mRNA level was measured by RT-qPCR in intestinal tissue in PAI-1(flx/flx) and C57BL/6J mice (n=8 mice per group) NS : non significant.

Supplementary Figure 2

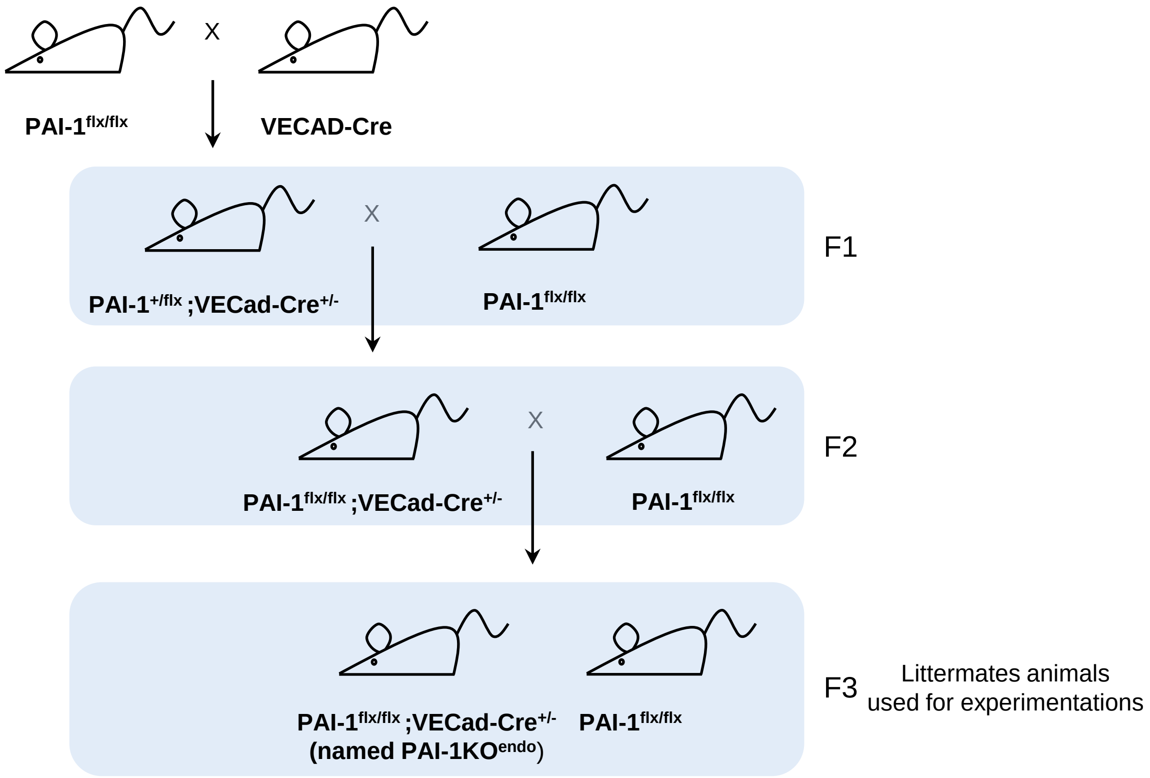

Supplementary Figure 2. Global breeding scheme for producing transgenic mice for experiments.

Supplementary Figure 3

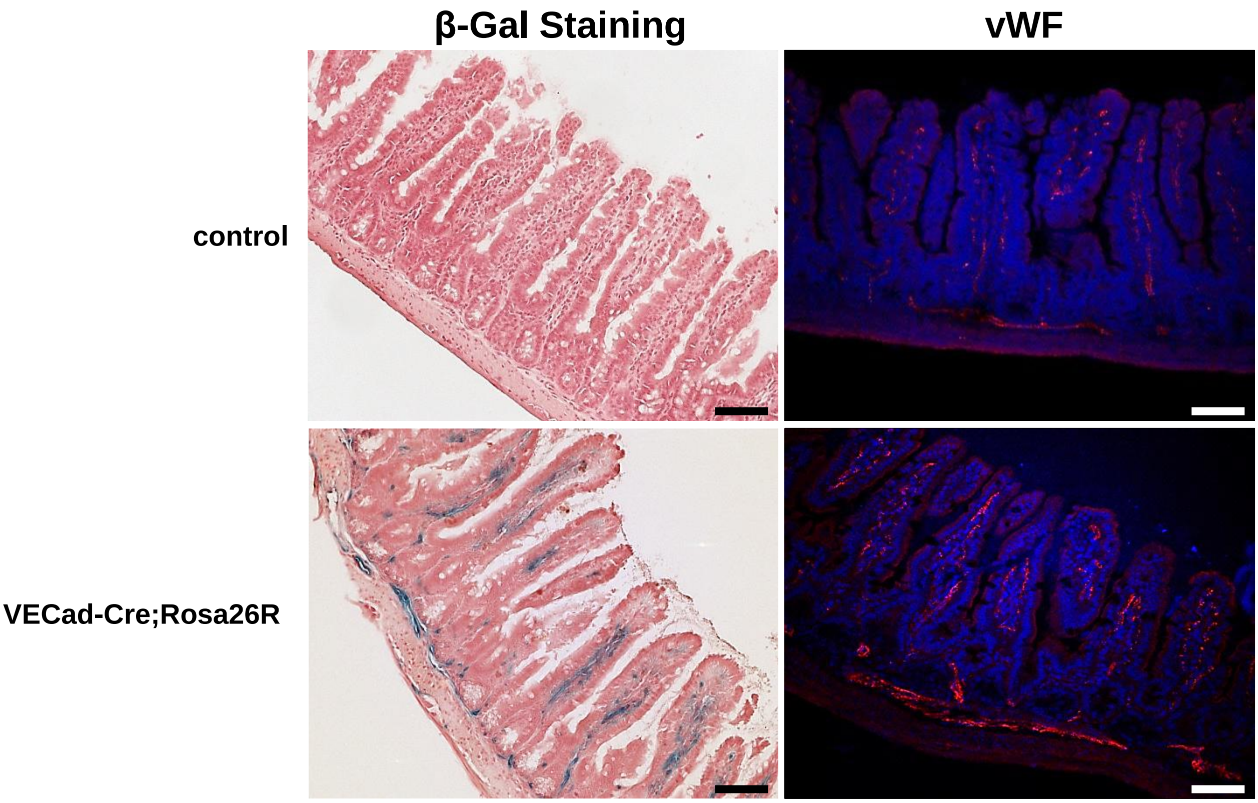

Supplementary Figure 3. Specific recombination events in endothelium in intestinal tissue. Recombination events were checked using ROSA26R reporter mice. LacZ staining (left) of gut from control ROSA26R (up) or VeCad-Cre/ROSA26R (down). Immunolabeling of von Willebrand factor is shown (right). Scale bar = 100 µm.

Supplementary Figure 4

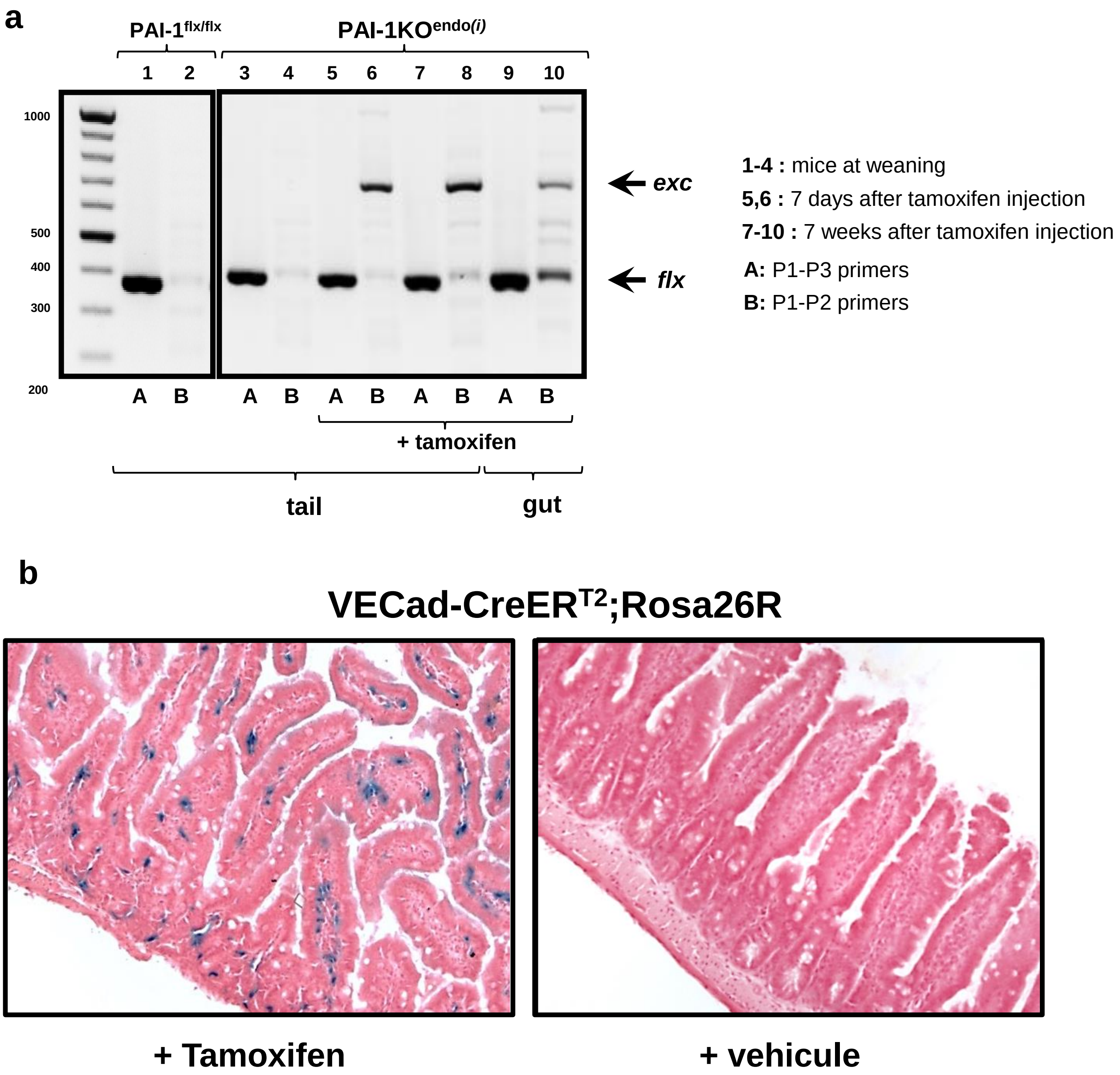

Supplementary Figure 4. (**a**) Genotype identification from DNA tail by PCR to discriminate PAI-1 floxed or excised allele in PAI-1KO$^{endo(i)}$ mice, 1 and 7 weeks after the first tamoxifen injection. The excised allele was also checked in gut. (**b**) LacZ staining of gut from control VECad-CreER^{T2}/ROSA26R 14 days after the first tamoxifen injection (left) or treated with vehicle solution (right).

Supplementary Figure 5

a

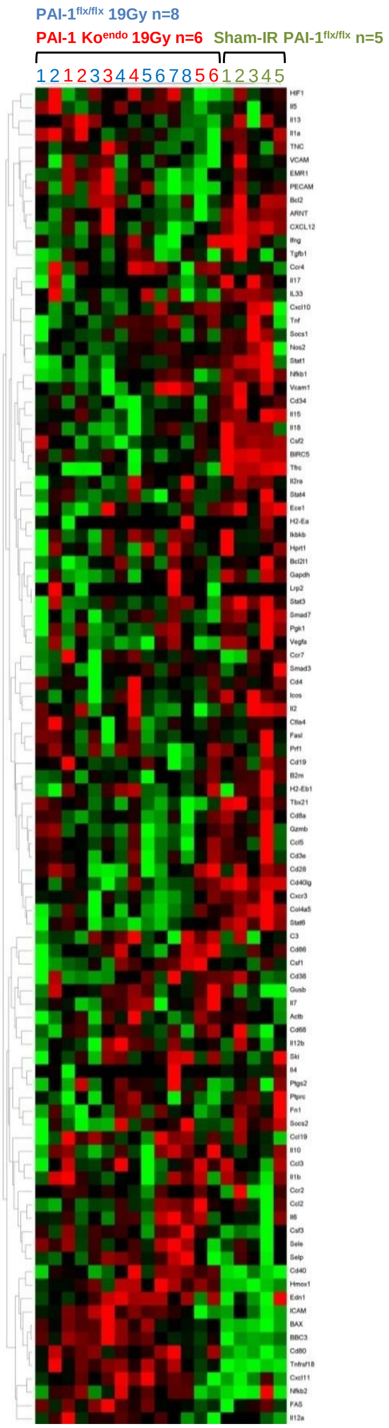

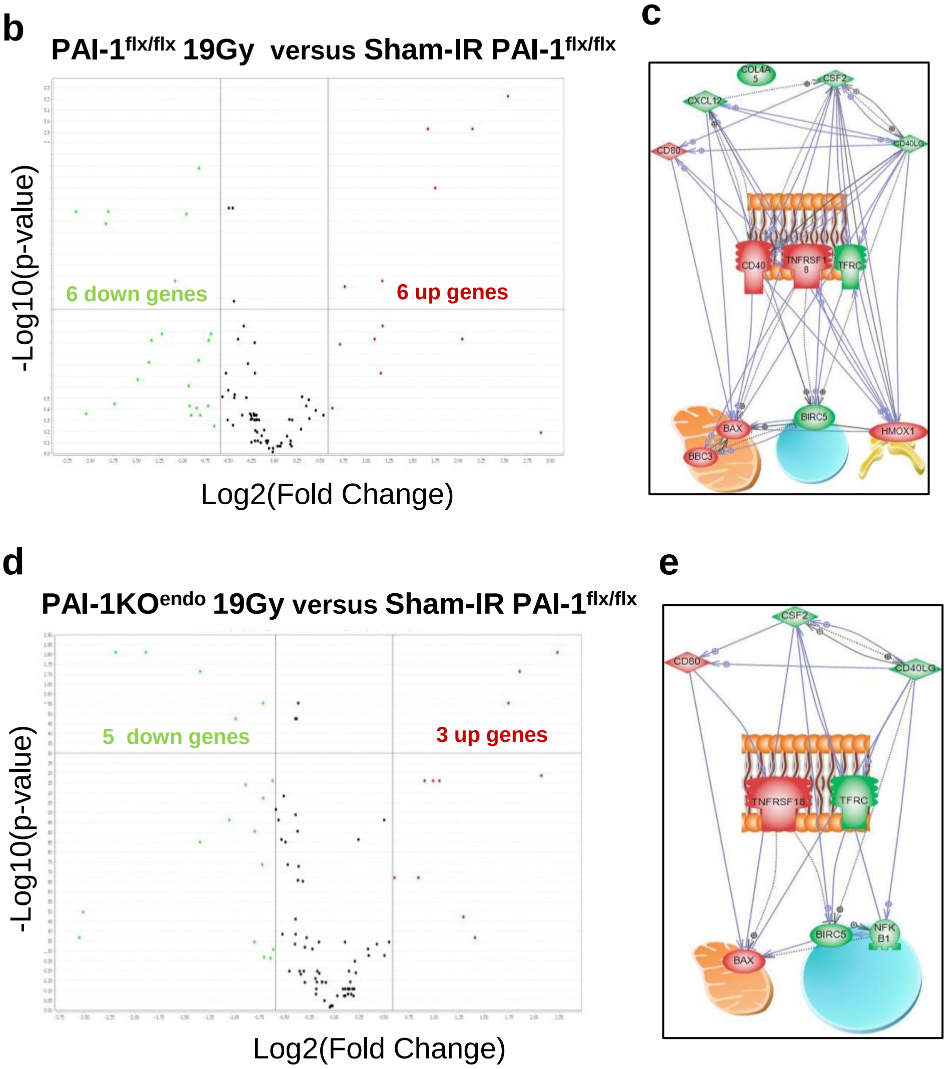

f

	5 hours after irradiation			
	PAI-1fl/fl 19 Gy vs Sham-IR PAI-1fl/fl		PAI-1KOendo vs Sham-IR PAI-1fl/fl	
**gene**	Fold change	P-value (FDR)	Fold change	P-value (FDR)
BAX	3,13	0,001	3,35	0,028
BBC3	3,31	0,003		
BIRC5	0,22	0,007	0,27	0,016
CD40	1,68	0,041		
CD40lg	0,28	0,007	0,50	0,034
CD80	4,40	0,002	3,61	0,019
CSF2	0,46	0,019	0,39	0,019
CXCL12	0,56	0,003		
Hmox1	2,24	0,033		
NFKB1			0,61	0,028
TFRC	0,28	0,007	0,22	0,016
TNFRSF18	5,73	0,001	4,70	0,016
Col4A5	0,51	0,009		

Supplementary Figure 5. Differential expression of inflammation/immune-related genes in the mouse intestine 5 hours after irradiation. (**a**) Hierarchical clustering analyses were performed and the results were visualized in the heat map. (**b-d**) Volcano plot analyses with a fold change boundary of 1.5 and an adjusted P-value ≤ 0.05 using the Benjamini-Hochberg false discovery rate. (**c-e**) Molecular pathways were build using Pathway Studio software based on differential expression of inflammation/immune-related genes in the mouse intestine 5 hours after irradiation. Pathways obtained in irradiated PAI-1$^{flx/flx}$ mice (**c**) and irradiated PAI-1KO^{endo} mice (**e**) compared with sham PAI-1$^{flx/flx}$ mice. (**f**) Table of fold changes and adjusted P-values of differentially expressed genes.

Supplementary Figure 6

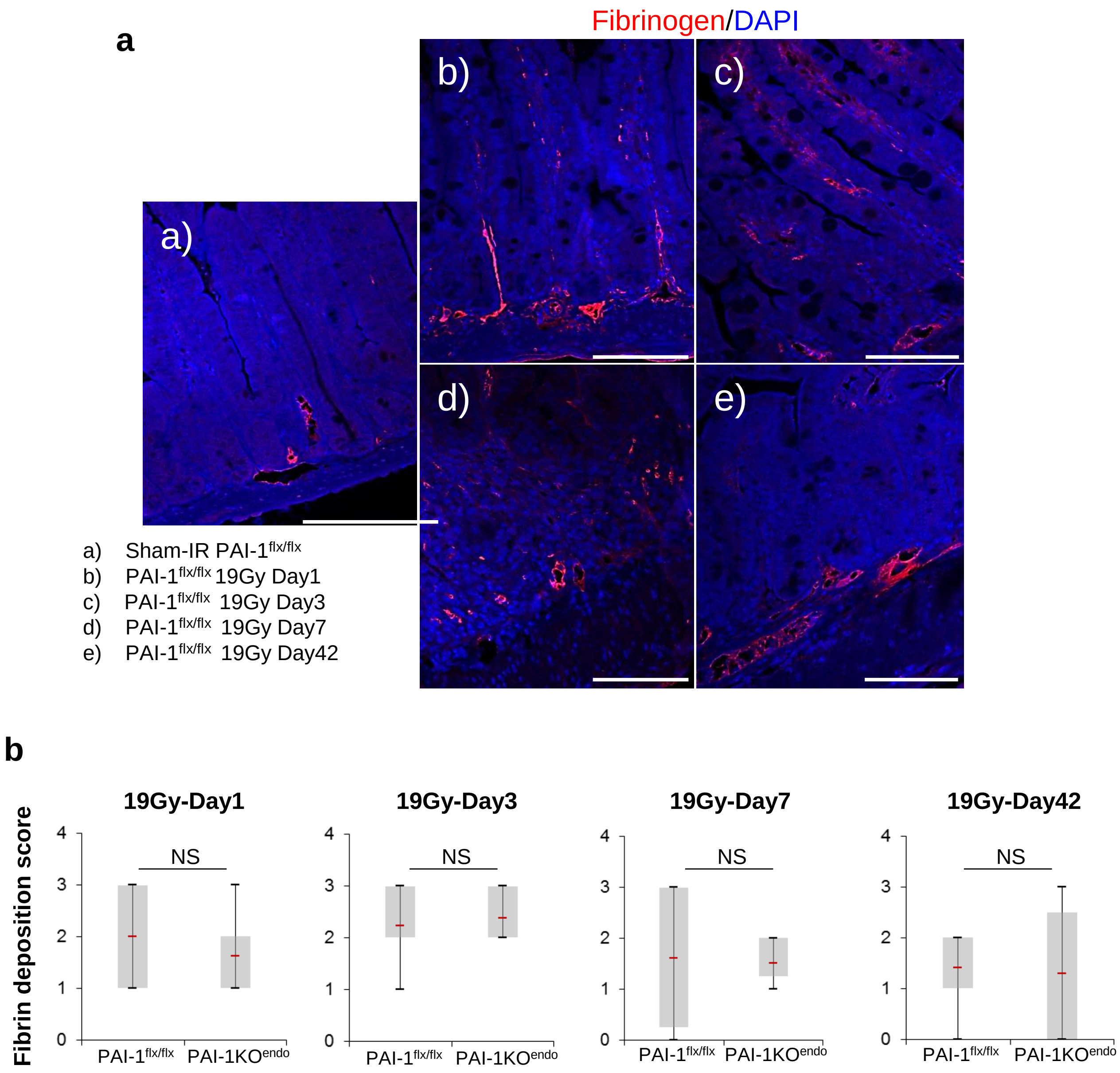

Supplementary Figure 6. Endothelial inactivation of PAI-1 does not affect the radiation-induced microvascular fibrin deposition (**a**) Fibrin deposition was visualized by immunolabeling with antibodies against fibrinogen/fibrin (red) and counterstaining with DAPI (blue). Representative images obtained in PAI-1$^{flx/flx}$ mice at 1, 3, 7 and 42 days after irradiation. (**b**) Fibrin deposition score in PAI-1$^{flx/flx}$ mice and PAI-1KO^{endo} mice 1 to 42 days after irradiation with n=7 to 10 mice per group. For all sham group mice (not shown) scores were 0.

Supplementary Figure 7

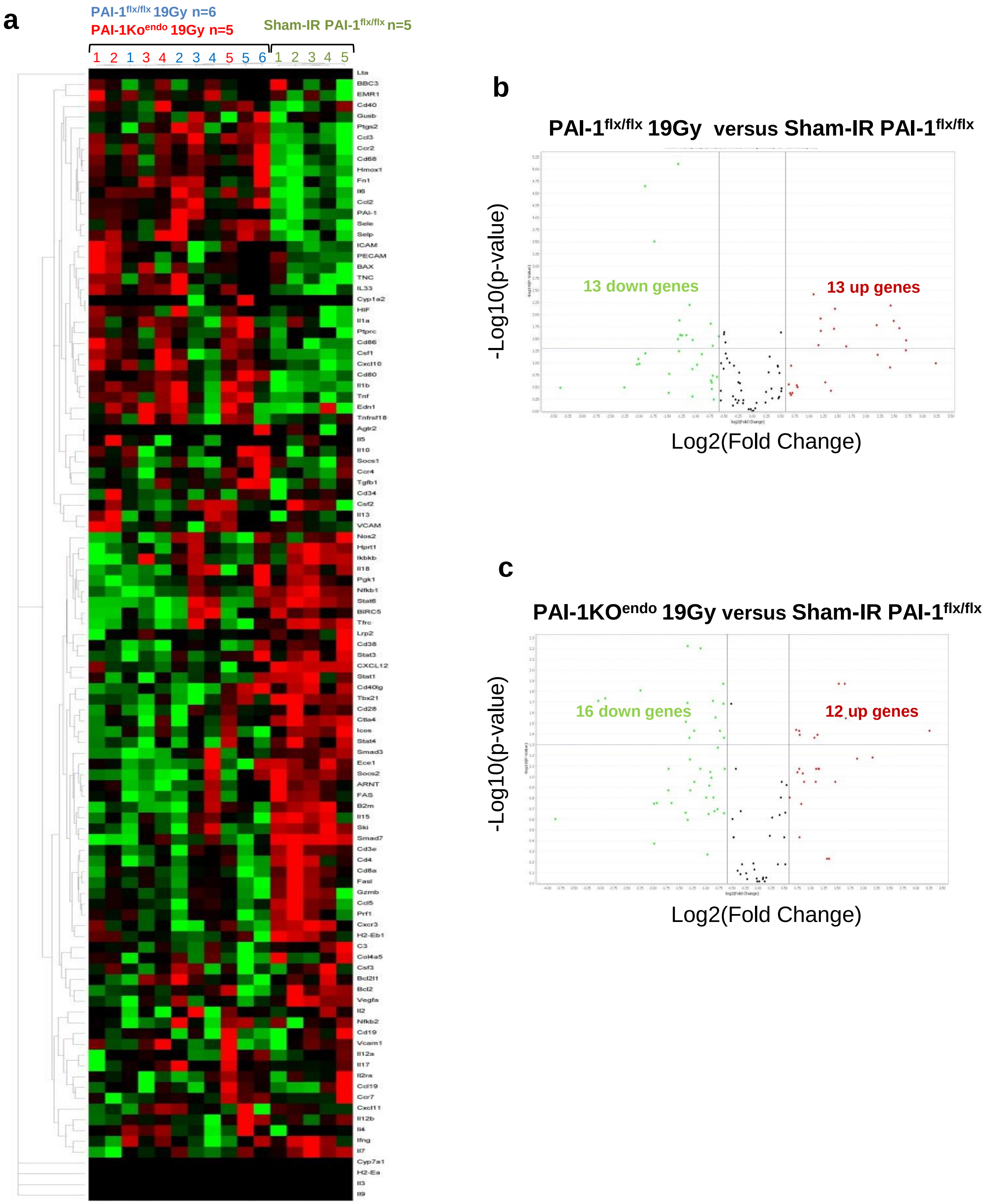

Supplementary Figure 7. Differential gene expression of inflammation/immune-related genes in the mouse intestine 3 days after irradiation. (**a**) Hierarchical clustering analyses were performed and results were visualized in the heat map. (**b-c**) Volcano plot analyses with a fold change boundary of 1.5 and an adjusted p-value ≤ 0.05 using the Benjamini-Hochberg false discovery rate.

Supplementary Figure 8

a **PAI-1$^{flx/flx}$ 19Gy versus Sham-IR PAI-1$^{flx/flx}$**

b **PAI-1KO^{endo} 19Gy Versus Sham-IR PAI-1$^{flx/flx}$**

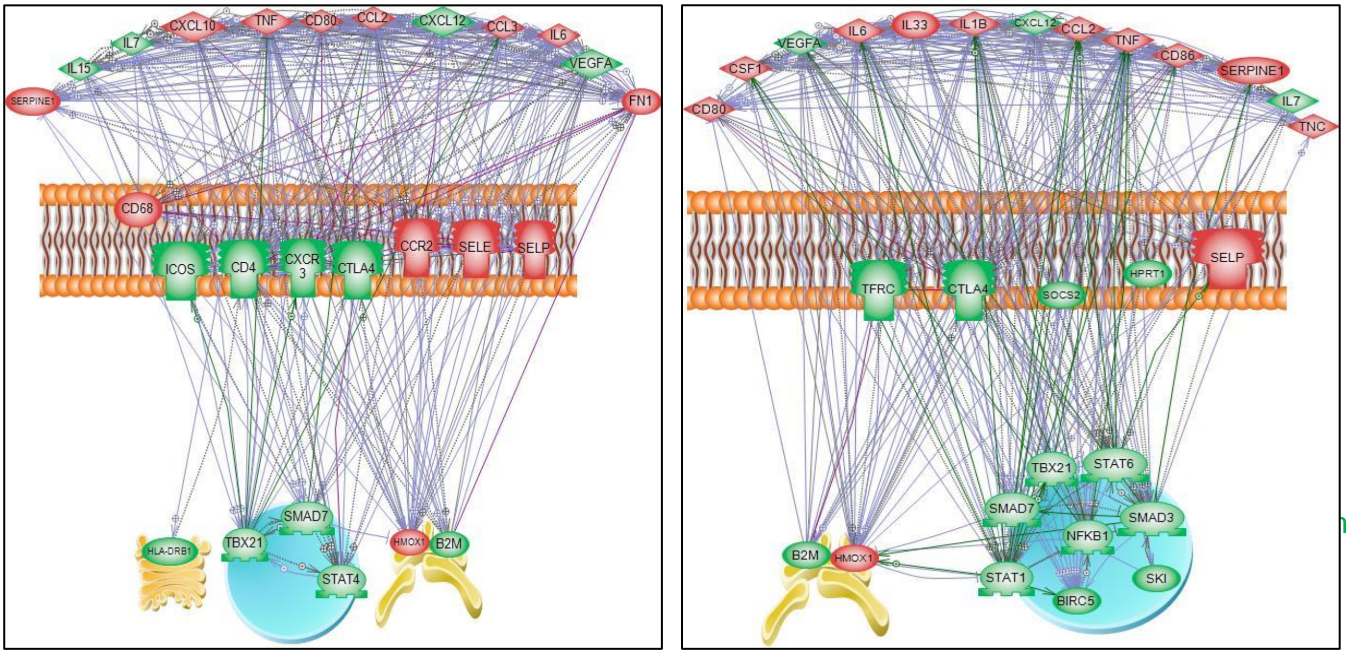

Supplementary Figure 8. Interactomes of the differentially expressed genes related to inflammation-immunity established using Pathway Studio software (mouse intestine 3 days after irradiation). Interactomes obtained in irradiated PAI-1$^{flx/flx}$ mice (**a**) and irradiated PAI-1KO^{endo} mice (**b**) compared with sham-IR PAI-1$^{flx/flx}$ mice. Red, up-regulated, green, down-regulated

Supplementary Figure 9

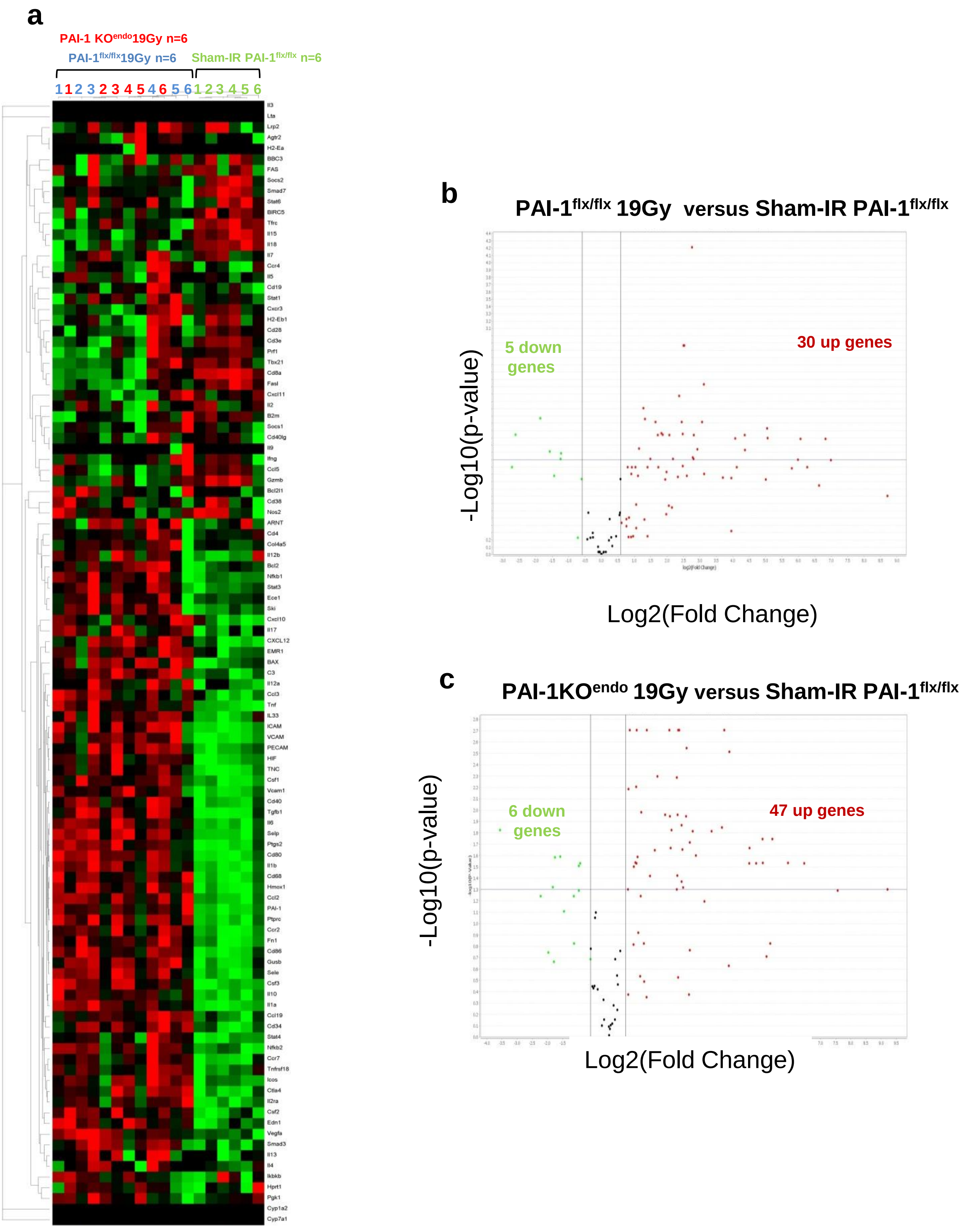

Supplementary Figure 9. Differential gene expression of inflammation/immune-related genes in the mouse intestine 7 days after irradiation. (**a**) Hierarchical clustering analyses were performed and results were visualized in the heat map. (**b-c**) Volcano plot analyses with a fold change boundary of 1.5 and an adjusted p-value ≤ 0.05 using the Benjamini-Hochberg false discovery rate.

Supplementary Figure 10

a **PAI-1$^{flx/flx}$ 19Gy versus Sham-IR PAI-1$^{flx/flx}$**

b **PAI-1KO^{endo} 19Gy versus Sham-IR PAI-1$^{flx/flx}$**

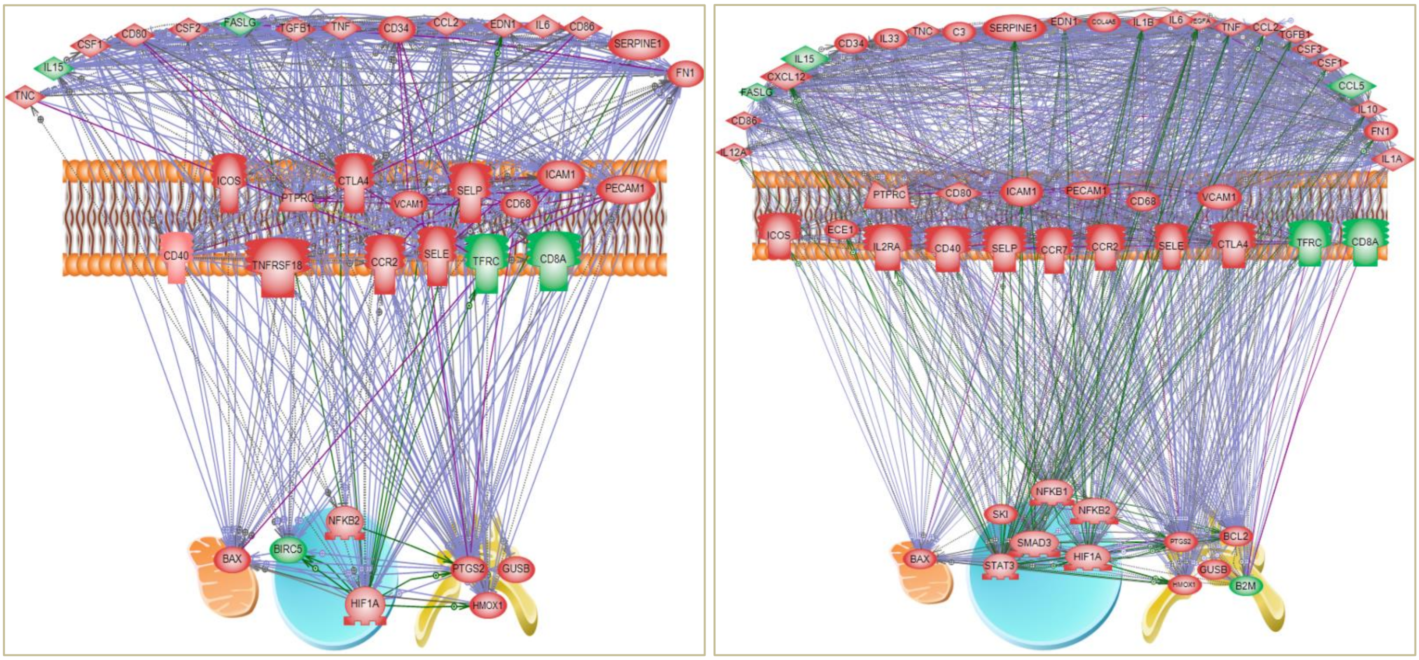

Supplementary Figure 10. Interactomes of the differentially expressed genes related to inflammation-immunity established using Pathway Studio software (mouse intestine 7 days after irradiation). Interactomes obtained in irradiated PAI-1$^{flx/flx}$ mice (**a**) and irradiated PAI-1KO^{endo} mice (**b**) compared with sham-IR PAI-1$^{flx/flx}$ mice. Red, up-regulated, green, down-regulated.

Supplementary Figure 11

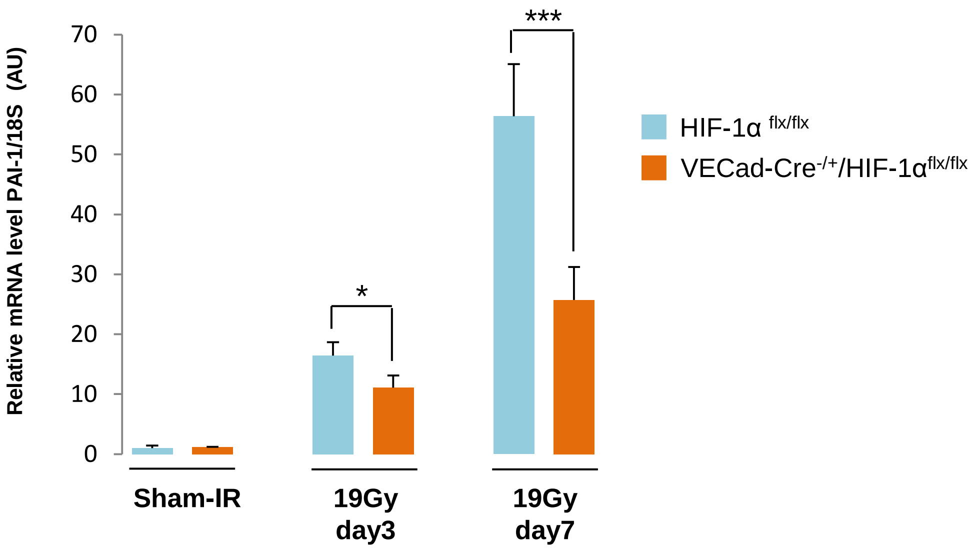

Supplementary Figure 11. HIF-1α endothelial deletion limits radiation-induced up-regulation of intestinal PAI-1 expression. Relative PAI-1 mRNA level was measured by RT-qPCR in intestinal tissue in HIF-1α $^{flx/flx}$ mice and VECad-Cre$^{+/-}$/HIF-1α$^{flx/flx}$ mice. Results are mean ± SEM with n= 6 to 8 mice per group. *, P<0.05 and ***, P<0.001.

Supplementary Figure 12

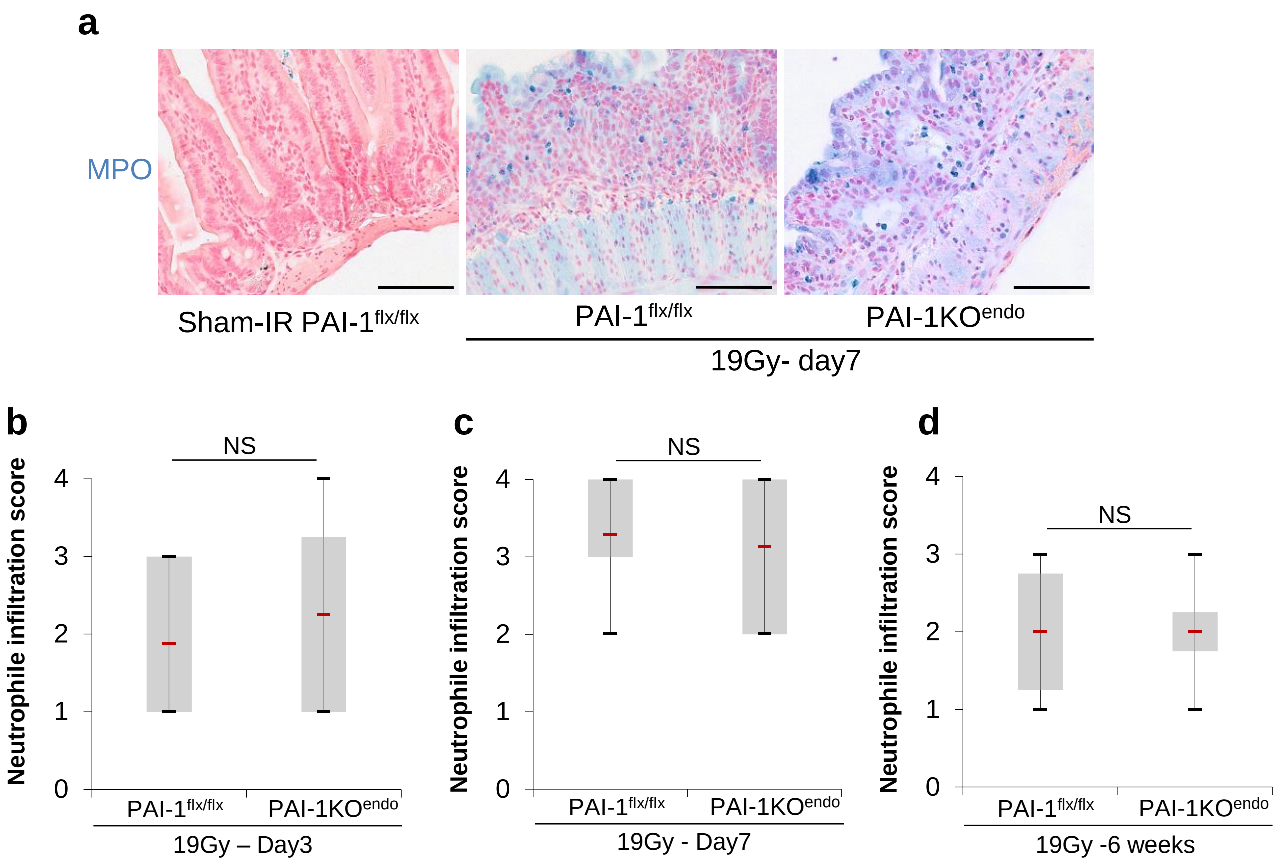

Supplementary Figure 12. Endothelial inactivation of PAI-1 does not affect the severity of neutrophil infiltrate after irradiation. (**a**) Representative labeling of neutrophils 7 days after irradiation. Neutrophils were immunolabeled using antibodies against myeloperoxidase (MPO) (blue) and slides were counterstained with nuclear fast red (pink). Scale bar = 100 µm. n= 5 for sham-IR PAI-1$^{flx/flx}$ mice, n=7 for PAI-1$^{flx/flx}$ 19 Gy mice, and n=8 for PAI-1KO^{endo} 19 Gy mice. Scores ranged from 0 (sham-IR not shown) to 4 (maximum neutrophil count) 3 days (**b**) 1 week (**c**) and 6 weeks after irradiation (**d**). NS, non-significant.

Supplementary Figure 13

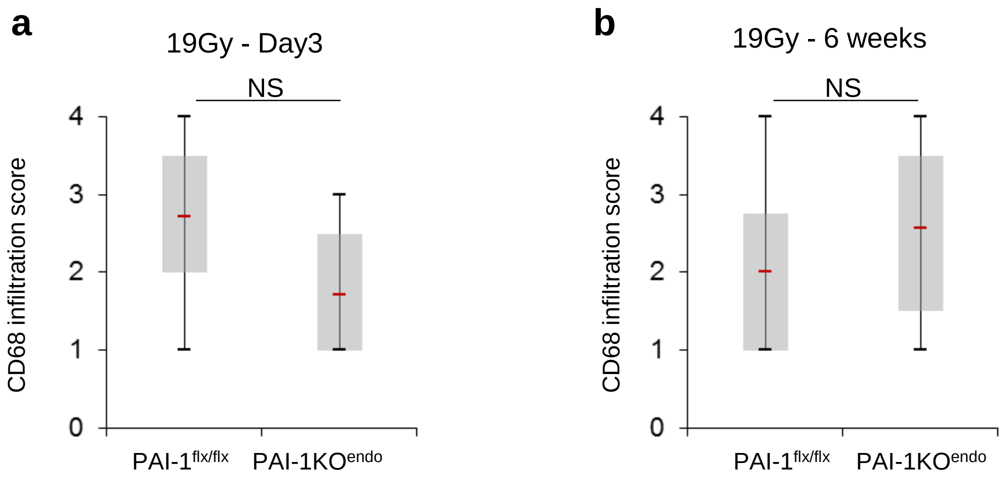

Supplementary Figure 13. Macrophage scoring (CD68-positive cells). Scores ranged from 0 (sham-IR) to 4 (maximum macrophage count) 3 days (**a**) and 6 weeks (**b**) after irradiation. n= 6 for PAI-$1^{flx/flx}$ sham-IR mice, n=8 for PAI-$1^{flx/flx}$ 19 Gy mice, and n=6 for PAI-1 KO^{endo} 19 Gy mice.

	3 days after irradiation			
	PAI-1$^{fl/fl}$ 19 Gy vs Sham-IR PAI-1$^{fl/fl}$		PAI-1KO^{endo} vs Sham-IR PAI-1$^{fl/fl}$	
**gene**	Fold change	P-value (FDR)	Fold change	P-value (FDR)
B2M	0,66	0,028	0,57	0,027
BIRC5			0,12	0,019
Ccl2	5,61	0,013	2,93	0,027
Ccl3	5,40	0,006		
Ccr2	2,30	0,012		
CD4	0,40	0,032		
CD68	2,74	0,007		
CD80	3,14	0,045	2,88	0,013
CD86			1,71	0,037
CSF1			1,65	0,036
CTLA4	0,30	0,000	0,38	0,030
CXCL10	2,30	0,021		
CXCL12	0,27	0,000	0,21	0,015
CXCR3	0,46	0,006		
FN1	2,71	0,014		
H2-Eb1	0,42	0,027		
Hmox1	4,56	0,016	3,16	0,028
HPRT1			0,60	0,037
ICOS	0,41	0,026		
IL15	0,44	0,027		
IL1B			9,53	0,037
IL33			3,12	0,013
Il6	6,01	0,019	5,31	0,023
IL7	0,61	0,044	0,43	0,037
NFKB1			0,55	0,019
PAI-1	9,38	0,045	3,74	0,023
Sele	6,53	0,034		
Selp	2,10	0,038	2,09	0,040
SKI			0,64	0,020
SMAD3			0,63	0,013
SMAD7	0,40	0,000	0,39	0,050
SOCS2			0,39	0,020
STAT1			0,58	0,050
STAT4	0,48	0,033		
STAT6			0,47	0,006
Tbx21	0,41	0,001	0,40	0,043
TFRC			0,15	0,018
TNC			1,73	0,040
TNF	2,24	0,040	2,18	0,040
VEGFA	0,61	0,001	0,63	0,040

	7days after irradiation			
	PAI-1$^{fl/fl}$ 19 Gy vs Sham-IR PAI-1$^{fl/fl}$		PAI-1KO^{endo} vs Sham-IR PAI-1$^{fl/fl}$	
**gene**	Fold change	P-value (FDR)	Fold change	P-value (FDR)
B2m			0,51	0,031
BAX	2,21	0,035	2,13	0,010
Bcl2			1,90	0,029
BIRC5	0,43	0,041		
C3			4,11	0,011
Ccl2	66,67	0,026	60,95	0,029
Ccl5			0,28	0,048
Ccr2	6,77	0,000	6,45	0,019
Ccr7			5,54	0,048
CD34	3,13	0,015	2,91	0,023
CD40	7,56	0,036	5,36	0,043
CD68	8,68	0,005	4,96	0,002
CD80	16,85	0,026	14,17	0,002
CD86	5,65	0,001	4,04	0,002
CD8a	0,16	0,023	0,08	0,015
Col4a5			1,57	0,050
CSF1	3,29	0,023	3,08	0,005
CSF2	5,44	0,015		
CSF3			590,21	0,050
Ctla4	5,55	0,022	4,79	0,050
CXCL12			3,68	0,011
Ece1	1,47	0,288	1,89	0,006
Edn1	3,65	0,023	4,16	0,022
Fasl	0,42	0,049	0,34	0,026
Fn1	8,38	0,015	7,41	0,025
Gusb	2,42	0,010	2,42	0,002
HIF	5,17	0,007	5,35	0,014
Hmox1	32,98	0,019	15,92	0,003
ICAM	6,86	0,047	5,93	0,011
Icos	2,81	0,049	2,60	0,038
Il10			13,47	0,014
Il12a			25,05	0,029
Il15	0,34	0,039	0,30	0,026
Il1a			42,54	0,018
Il1b			88,19	0,029
Il2ra			4,85	0,038
IL33			4,26	0,015
Il6	113,27	0,026	189,65	0,050
NFKB1			1,92	0,002
NFKB2	2,51	0,014	1,96	0,026
PAI-1	63,21	0,050	34,24	0,029
PECAM	4,54	0,049	4,81	0,005
Ptgs2	20,67	0,037	29,41	0,029
Ptprc	5,75	0,001	4,89	0,011
Sele	20,56	0,023	25,25	0,022
Selp	33,32	0,025	34,02	0,018
SKI			1,64	0,002
SMAD3			1,80	0,031
STAT3			1,59	0,007
TFRC	0,28	0,014	0,53	0,029
Tgfb1	6,98	0,049	5,46	0,022
TNC	7,02	0,023	10,65	0,015
TNF	17,41	0,043	6,89	0,015
TNFRSF18	3,54	0,022		
VCAM1	4,18	0,023	5,08	0,002
VEGFA			1,86	0,029

Supplementary Table 1: List of differentially expressed genes with corresponding fold changes and adjusted P-values using the Benjamini-Hochberg false discovery rate in intestinal tissue 3 and 7 days after irradiation in irradiated PAI-1$^{flx/flx}$ mice and irradiated PAI-1KO^{endo} mice compared with Sham-IR PAI-1$^{flx/flx}$ mice. Red indicates genes up-regulated and green genes down-regulated after irradiation.

Day 7 post irrdadiation		
GO TERM (BIOLOGICAL PROCESS)	ENTITIES (n)	P-VALUE
PAI-1$^{flx/flx}$		
cell adhesion	SELE,SELP,CD34,VCAM1,ICAM1,FN1,PECAM1,CCL2,TNC **(9)**	0,013
positive regulation of apoptotic process	PTGS2,HIF1A,PTPRC,IL6,TGFB1,TNF,FASLG,BAX,CTLA4 **(9)**	0,025
modulation by virus of host morphology or physiology	TFRC,VCAM1,ICAM1,TGFB1,BAX,CD80,CD86,CCR2 **(8)**	0,003
angiogenesis	HMOX1,PTGS2,HIF1A,FN1,CCL2,IL15,CCR2,SERPINE1 **(8)**	0,007
leukocyte migration	SELE,SELP,CD34,ICAM1,FN1,PECAM1,TNF **(7)**	0,002
platelet activation	SELP,CD40,FN1,PECAM1,IL6,TGFB1,SERPINE1 **(7)**	0,007
response to wounding	FN1,IL6,TNC,TGFB1,BAX,CCR2 **(6)**	0,001
extracellular matrix organization	NFKB2,FN1,TNC,TGFB1,TNF,SERPINE1 **(6)**	0,006
positive regulation of NF-kappaB transcription factor activity	NFKB2,CD40,ICAM1,IL6,TGFB1,TNF **(6)**	0,039
platelet degranulation	SELP,FN1,PECAM1,TGFB1,SERPINE1 **(5)**	0,017
cell-cell adhesion	SELP,CD34,VCAM1,ICAM1,ICOS **(5)**	0,017
leukocyte cell-cell adhesion	SELE,SELP,PTPRC,VCAM1,ICAM1 **(5)**	0,043
leukocyte tethering or rolling	SELE,SELP,VCAM1,TNF **(4)**	0,012
heterophilic cell-cell adhesion	SELE,SELP,VCAM1,ICAM1 **(5)**	0,012
response to amino acid	ICAM1,CCL2,IL6,EDN1 **(4)**	0,012
positive regulation of MAP kinase activity	CD40,EDN1,TGFB1,TNF **(4)**	0,044
myeloid dendritic cell differentiation	CSF2,TGFB1,CD86 **(3)**	0,037
osteoclast differentiation	TFRC,CSF1,TNF **(3)**	0,037
positive regulation of MAPK cascade	PTPRC,IL6,CCR2 **(3)**	0,037
regulation of vascular endothelial growth factor production	CCL2,IL6,CCR2 **(3)**	0,037
negative regulation of blood coagulation	CD34,EDN1,SERPINE1 **(3)**	0,037
regulation of angiogenesis	HMOX1,IL6,SERPINE1 **(3)**	0,037
positive regulation of odontogenesis	CD34,EDN1,TGFB1 **(3)**	0,037
negative regulation of fat cell differentiation	IL6,TGFB1,TNF **(3)**	0,037
cellular iron ion homeostasis	HMOX1,HIF1A,TFRC **(3)**	0,037
regulation of cell migration	PECAM1,TGFB1,CCR2 **(3)**	0,037
response to ionizing radiation	VCAM1,ICAM1,BAX **(3)**	0,037
PAI-1KO^{endo}		
response to hypoxia	HMOX1,HIF1A,SMAD3,TFRC,VCAM1,ICAM1,CCL2,VEGFA,EDN1,TGFB1,TNF,BCL2,IL1B,IL1A,CCR2,ECE1 **(16)**	0,029
negative regulation of cell proliferation	HMOX1,PTGS2,SMAD3,STAT3,IL6,TGFB1,TNF,BCL2,BAX,IL1B,IL1A,SKI **(12)**	0,001
aging	NFKB2,TFRC,VCAM1,CCL2,CCL5,IL15,IL6,IL10,TGFB1,BCL2,CD86,IL1B **(12)**	0,028
positive regulation of apoptotic process	PTGS2,HIF1A,IL2RA,PTPRC,IL6,TGFB1,TNF,FASLG,BAX,CTLA4,IL1B **(11)**	0,046
response to glucocorticoid	PTGS2,FN1,CCL2,CCL5,IL6,IL10,TNF,BCL2,IL1B,C3,SERPINE1 **(11)**	0,002
cell proliferation	STAT3,IL2RA,CD34,VEGFA,CSF1,TGFB1,TNF,BCL2,BAX,IL1A,SKI **(11)**	0,046
positive regulation of angiogenesis	HMOX1,HIF1A,CD34,CCL5,VEGFA,IL1B,IL1A,C3,SERPINE1 **(9)**	0,008
modulation by virus of host morphology or physiology	STAT3,TFRC,VCAM1,ICAM1,TGFB1,BAX,CD80,CD86,CCR2 **(9)**	0,008
angiogenesis	HMOX1,PTGS2,HIF1A,FN1,CCL2,VEGFA,IL15,CCR2,SERPINE1 **(9)**	0,026
leukocyte migration	SELE,SELP,CD34,ICAM1,FN1,PECAM1,TNF,IL1B **(8)**	0,003
platelet activation	SELP,CD40,FN1,PECAM1,VEGFA,IL6,TGFB1,SERPINE1 **(8)**	0,016
positive regulation of NF-kappaB transcription factor activity	NFKB1,NFKB2,CD40,ICAM1,IL6,TGFB1,TNF,IL1B **(8)**	0,016
blood coagulation	SELE,SELP,FN1,PECAM1,VEGFA,TGFB1,C3,SERPINE1 **(8)**	0,046
positive regulation of smooth muscle cell proliferation	HMOX1,PTGS2,HIF1A,CCL5,VEGFA,IL6,EDN1,TNF **(8)**	0,046
extracellular matrix organization	NFKB2,FN1,COL4A5,TNC,TGFB1,TNF,SERPINE1 **(7)**	0,006
response to organic substance	PTGS2,STAT3,TFRC,IL10,TGFB1,BCL2,IL1A **(7)**	0,030
wound healing	SMAD3,FN1,PECAM1,TGFB1,IL1B,IL1A,SERPINE1 **(7)**	0,030
platelet degranulation	SELP,FN1,PECAM1,VEGFA,TGFB1,SERPINE1 **(6)**	0,013
cell-cell adhesion	SELP,CD34,VCAM1,ICAM1,BCL2,ICOS **(6)**	0,013
response to copper ion	NFKB1,TFRC,ICAM1,BCL2,BAX,IL1A **(6)**	0,013
response to wounding	FN1,IL6,TNC,TGFB1,BAX,CCR2 **(6)**	0,013
positive regulation of peptidyl-serine phosphorylation	VEGFA,CSF3,IL6,TGFB1,TNF,BCL2 **(6)**	0,013
hemopoiesis	CD34,CSF1,IL10,BCL2,CCR2 **(5)**	0,028
positive regulation of MAP kinase activity	CD40,VEGFA,EDN1,TGFB1,TNF **(5)**	0,028
positive regulation of interleukin-6 production	IL6,TNF,IL1B,IL1A,IL33 **(5)**	0,028
response to heat	CCL2,IL6,BCL2,IL1B,IL1A **(5)**	0,028

Supplementary Table 2: Gene ontology (GO) enrichment analyses (biological processes) corrected for non-genome-wide analyses. Pathway Studio software was used to analyze the list of up- and down-regulated genes at any time post-irradiation in irradiated PAI-1$^{flx/flx}$ mice and PAI-1KO^{endo} mice compared with Sham PAI-1$^{flx/flx}$ mice. Analyses were adjusted to the initial list of measured genes (n=106). The table displays GO terms with a P-value <0.05 at day 7 post-irradiation. For each GO term, the number and name of the entities as well as the P-value are given.
